# Combinatorial Use of Reference Electrodes and DRT for Disentangling AEM Electrolyzer Losses

**DOI:** 10.1021/acs.energyfuels.5c01799

**Published:** 2025-08-15

**Authors:** Suhas Nuggehalli Sampathkumar, Thomas Benjamin Ferriday, Samaneh Daviran, Hamza Moussaoui, Philippe Aubin, Khaled Lawand, Mounir Mensi, Pascal Alexander Schouwink, Albert Taureg, Vanja Subotić, Arthur Paul Lucien Thévenot, Fabio Dionigi, Peter Strasser, Jan Van Herle

**Affiliations:** † Group of Energy Materials, 27218 École polytechnique fédérale de Lausanne (EPFL), Rue de l’Industrie 17, Sion, Valais 1951, Switzerland; ‡ Centre for Materials Science and Nanotechnology, 6305 University of Oslo, Gaustadalléen 21, Oslo 0349, Norway; § X-Ray Diffraction and Surface Analytics Facility, 150727 École polytechnique fédérale de Lausanne (EPFL), Rue de l’Industrie 17, Sion 1951, Switzerland; ∥ ENAC Interdisciplinary Platform for X-ray micro-tomography (PIXE), École polytechnique fédérale de Lausanne (EPFL), Station 18, Lausanne, Vaud 1015, Switzerland; ⊥ Institute of Thermal Engineering, 27253 Graz University of Technology, Inffeldgasse 25/B, Graz 8010, Austria; # Department of Chemistry, 26524 Technical University Berlin, Straße des 17, Juni 124, Berlin 10623, Germany

## Abstract

Anion exchange membrane water electrolyzers (AEMWEs) offer a promising alternative to proton exchange membrane (PEM) electrolyzers, leveraging non-precious-metal catalysts and alkaline electrolytes for cost reduction. However, challenges persist in achieving long-term durability, high current densities, and stable membrane performance. While previous studies have examined AEM development, a comprehensive structural-electrochemical analysis of AEMWE components under prolonged operation remains limited. This study presents a detailed structural and electrochemical characterization of a commercial AEMWE, where its full-cell performance was matched with the intrinsic half-electrode performance through the use of dual reference electrodes. The electrochemical analysis was supported by a thorough tomographic and spectroscopic investigation of each electrode, thereby providing for the first time a complete materials analysis of the commercial NiFeO_x_ anode and Raney nickel cathode. Electrochemical characterization using LSV, EIS, and a dual reference electrode setup revealed full-cell performance of 1.0 A cm^–2^ at 2.2 V (ambient) and 1.1 A cm^–2^ at 2.0 V (60 °C), with an HHV efficiency of 74.5% at 1.0 A cm^–2^. Long-term operation over 1000 h at 1.0 A cm^–2^, 60 °C, in 1.0 M KOH resulted in a substantial polarization resistance increase beyond 230 h, despite an unexpected continuous improvement in MEA performance due to membrane degradation. DRT analysis, coupled with reference electrode studies, was critical in isolating losses. Low-frequency peaks (1.5–25 Hz) were linked to bubble formation, while intermediate-frequency (50–2000 Hz) and high-frequency (>2000 Hz) processes corresponded to charge transfer and ionic transport. The NiFeO_x_ anode exhibited better charge transfer, whereas the Raney nickel cathode showed higher polarization resistance.

## Graphical abstract

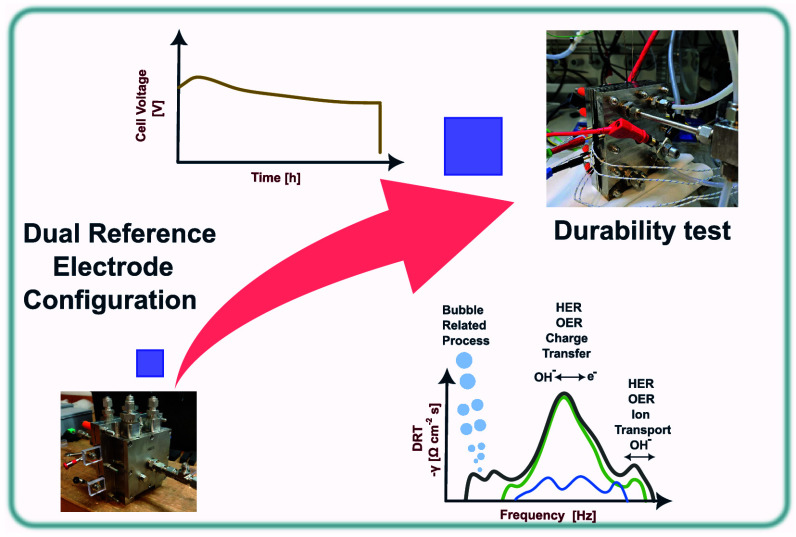

## 1 Introduction

Fueled by the green energy transition, hydrogen technology has taken tremendous steps in the last decades.^1,2^ Green hydrogen has become more available through water electrolyzer (WE) technologies such as the alkaline WE (AWE) and the proton exchange membrane WE (PEMWE). However, both technologies face issues, namely, the potent supporting electrolyte employed in AWEs (30 wt % KOH), gas crossover through their separators, and low current densities (≈ 0.4 A cm^–2^), which affect balance-of-plant, gas purity, and performance, respectively.^1–3^ For PEMWEs, expensive titanium hardware and platinum group metal (PGM) catalyst materials limit their potential to scale to the necessary level.^4,5^ A possible answer to these issues lies in the comparatively recent anion exchange membrane WE (AEMWE) technology.^2,6^

AEMWEs emerge as a combination of the AWE and the PEMWE, wherein an alkaline counterpart to the ion-conducting membrane featured in PEMWEs is employed in a zero-gap AWE to great effect. This addition increases current density (2.0 vs ≈ 0.4 A cm^–2^), lowers gas crossover, and requires a less potent supporting electrolyte than its predecessor (1.0 M KOH vs 30 wt % KOH ≈ 5.35 M KOH).^4–6^ Moreover, the AEMWE retains the original benefits the AWE held over the PEMWE in the ability to use inexpensive hardware (stainless steel vs titanium), abundant catalyst materials (Ni and Fe vs Pt and Ir), and avoids the use of environmentally hazardous perfluorinated membranes such as Nafion.^6^

AEMWE technology is envisioned to surpass PEMWE; however, the former is currently dependent on a supporting electrolyte to rival PEM performances, as their efficacy with deionized water as a reactant is notably inferior to that with KOH. However, great progress has been made in recent years with advancing deionized water-fed AEMWEs.^7,8^

Today, AEM technology has progressed to where small kW-size units are commercially available. Individual electrolyzer components are also commercialized, including the gas diffusion layer, micro porous layer, anode/cathode electrodes, and anion exchange membranes and ionomers.^6,9^ The utility of such commercial materials is their use in creating benchmarks against which the performance of future experimental materials or techniques may be compared.

Development of hydrogen technologies will be further accelerated by decoupling the performance of both commercial and experimental materials through establishing their weakest link. Decoupling the total overpotential yields profound insight which can be employed to reforge weak links, thereby improving material efficiency/longevity. Correctly decoupling overpotentials is tricky, but it is surmountable through engineering novel test cells with careful reference electrode placement, combined with innovative data analysis techniques. Existing efforts in the literature typically show a type of reference electrode being placed between anode and cathode either by extending the membrane^10–12^ or using a pseudo reference, i.e., adding a separate internal Pt ring reference into the membrane (typically by sandwiching two membranes together).^13–15^

Distribution of relaxation times (DRT) is an innovative type of impedance analysis technique that is well established for high-temperature hydrogen technologies.^16,17^ Investigating the frequency DRT contributes to dispelling ambiguity around various WE processes such as the transfer of electrical charge, catalytic charge, mass, etc. This technique has spread and is now a staple of many impedance-based investigations for both single reactions,^18,19^ single cells^20–24^ and stacks.^16,17,25,26^

Thus, combining both novel cell design and DRT analysis would appear as a powerful approach to clarify uncertainties regarding AEMWE performance limitations, such that new materials can be developed. Exemplified utilizing tried-and-tested commercial materials, we show in this paper the strengths of this approach. To cover all bases, we initially completed a thorough spectroscopic, tomographic, and electrochemical analysis of the current state-of-the-art commercial AEMWE materials. This analysis underscored the importance of verifying the material composition of the commercial materials. Most importantly, we show that a complete decoupling of overpotentials related to ohmic, kinetic, and mass transport is possible in an AEMWE by utilizing a novel dual reference electrode system together with DRT interpretation.

This allowed us to link the performance of intermediate-scale AEMWE cells (*A* = 25 cm^2^) to the fundamental three-electrode performance of their two electrodes, thereby dispelling the ambiguity of charge transfer and polarization losses in AEMWEs. Furthermore, we also report a 1000+ hour stability test of the intermediate-scale single-cell (60 °C, 1.0 A cm^–2^), where in situ impedance measurements allow us to attribute changes in performance to internal mechanisms such as initial break-in, OER/HER kinetic improvements, or deactivation.

This method leaves no ambiguity around the limiting factors of a AEMWE. A wide-scale implementation of this novel cell configuration will clearly reveal performance-limiting aspects, thereby accelerating the development of future experimental materials.

## 2 Experimental Procedure

Experimental procedures are divided between spectroscopic and electrochemical characterization of the electrode materials as shown in Figure 1a,b.

**Figure 1:**
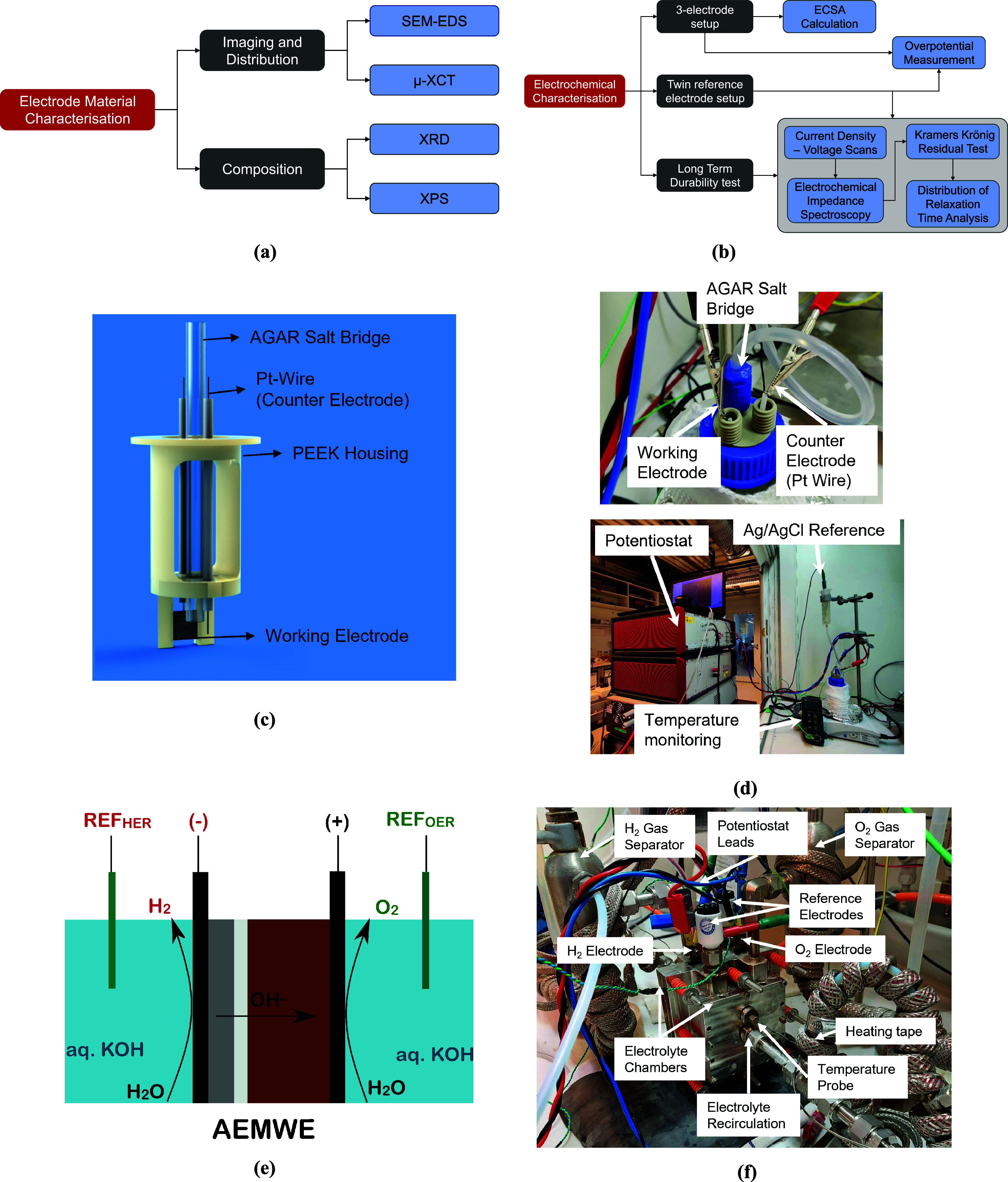
Experimental study order with (a) material characterization followed by (b) electrochemical characterization of the lab-scale materials and the complete MEA, and the full-scale, long-term test. Lab-scale materials were tested in the three-electrode setup schematic in (c), whose embodiment was created and is shown in (d). Similarly for the full cell, (e) shows the full-cell schematic with the placement of the reference electrodes and (f) represents the actual setup with the appropriate measurement and control possibilities.

### 2.1 Characterization of Electrode Materials

X-ray diffraction (XRD) measurements of the commercial electrodes were performed in a Bragg–Brentano configuration for angles between 5 and 120° using a D8 Advance (Bruker) diffractometer to quantify crystallography and bulk structure. The visual interpretation and bulk quantification of the catalyst distribution were established through scanning electron microscopy (SEM) using a Thermo Fisher Scientific Teneo equipped with Trinity and Everhart-Thornley electron detectors and energy-dispersive X-ray analysis (EDX) through use of the Bruker XFlash 6–30 silicon drift detector. X-ray photoelectron spectroscopy (XPS) measurements were carried out in a Kratos Axis Supra using a monochromatic Kα X-ray line from an aluminum anode. The pass energy was set to 40 eV with a step size of 1.0 eV for the survey spectra, and the core-level spectra employed a pass energy of 20 eV and a step size of 0.15 eV.

The porosity, effective conductivity, and diffusivity of both anodic and cathodic porous transport layers (PTLs) were identified using X-ray tomographic microscopy (XTM) through a RX-Solutions Ultratom X-ray microcomputed tomography scanner. A 0.22 mm copper filter was placed in front of a LaB_6_ cathode with a 160 kV microfocus X-ray source (Hamamatsu) to suppress beam hardening effects. A sample geometry of 10 × 2 × 0.6 mm^3^ was analyzed with a voxel cube size of 1.0 μm^3^. The acquisition parameters were set to 80 kV and 140 μA to record 1632 projections over 360° at 3 frames per second, with a frame averaging set to 6, effectively requiring 54 min per scan.

A Varex PaxScan 2530HE flat panel detector (2176 × 1792 pixels) was used to capture the transmitted X-rays. The projections were processed with the RX-Solutions X-act software, using a filtered back-projection method. Post-processing of the tomographic image was carried out based on the methodology adopted by Moussaoui et al.^27^ to identify porosity, connectivity, effective diffusivity, and conductivity. The effective diffusivity and conductivity are defined as ratios of bulk to substrate quantities, as shown below in eqs 1 and 2.

1 $$\epsilon =\frac{\epsilon_{\mathrm{bulk}}}{\epsilon_{\mathrm{pore}}}$$

2 $$\sigma =\frac{\sigma_{\mathrm{bulk}}}{\sigma_{\mathrm{fibre}}}$$

where ϵ_bulk_, ϵ_pore_ and σ_bulk_, σ_fiber_ are the diffusivity and conductivity of the bulk and the pore or fiber, respectively.

### 2.2 MEA Fabrication

The commercial non-PGM electrodes marketed as anodes and cathodes for water electrolyzers were purchased from Dioxide Materials. Their original conception was briefly described by Liu et al.,^9^ where NiFe_2_O_4_ on SS316L fiber paper served as the anode and NiFeCo deposited on nickel fiber paper was used as a cathode. Our materials analysis has conclusively shown that the cathode catalyst was not NiFeCo, but Raney nickel. Both electrodes were created through the catalyst-coated substrate (CCS) method,^6^ using Nafion perfluorinated resin solution as binder for both electrodes.

Several electrode sizes are featured in this paper, namely standard lab-size 1.0 cm^2^ electrodes used for three-electrode experiments, while considerably larger 25 cm^2^ electrodes were employed for long-term durability tests. These electrodes were electrically separated by a Sustainion X37–50 RT anion exchange membrane, also purchased from Dioxide Materials. The membrane was activated by following the standard Sustainion protocol of 24 h immersion in a 1.0 M KOH solution (prepared with deionized water), before being placed between the electrodes for electrochemical analysis.^9^ While there are several advanced methods for assembling an MEA,^6^ these components were simply sequentially assembled such that the subsequent MEA performance was contingent on the efficacy of each component rather than MEA optimization and engineering. Moreover, this also limited performance differences between three-electrode and full-cell setups, allowing simple assignment of full-cell performance limitations to three-electrode fundamentals.

### 2.3 Experimental Setup

#### 2.3.1 Three-Electrode Setup

Electrode kinetics were first studied using a three-electrode setup, as shown in Figure 1c,d, similar to our previous work.^28,29^ The PEEK body was designed to ensure a fixed distance between the working electrode (WE), the reference electrode, and the counter electrode (CE). A platinum wire was used as the counter electrode, while a Ag/AgCl (3.0 M KCl) reference electrode was connected through a salt bridge as initially described in our previous paper.^30^ Similar to the usual three-electrode setup,^31^ the reference electrode was placed between the WE and the CE.

#### 2.3.2 Dual Reference Electrode Setup

The commercial MEA was assembled and placed into an in-house-created housing (SS316L) accommodating the reference electrodes as shown in the schematic Figure 1e. The cathode and anode were placed next to each of their NiP_3_-coated SS316L current plate. The current plates were machined such that pockets allowed for efficient transfer of liquid and gas between the chamber and their respective electrodes. Uniform current distribution in the plates was achieved by the placement of nickel foam prior to the catalyst-coated substrates.

Low-impedance Hydroflex (Gaskatel) reversible hydrogen electrodes were necessary to quantify the HER and OER overpotentials in the multireference electrode setup. The Hydroflex RHE features a capillary that ensures equilibrium between the electrolyte solution near the sensor and the working electrode. This design eliminates the need to compensate for pH variations, allowing potentials to be directly calculated as overpotentials, see eqs S10 and S11. The RHE reference electrodes were immersed overnight in the working electrolyte (1.0 M KOH) to ensure proper filling of the capillary.

Additionally, the setup ensured equidistant placement of both RHEs. Supporting electrolyte was supplied to the cell using magnetic pumps attached to a heated high-volume reservoir, and the connecting pipes were covered in heating tape to minimize temperature gradients. The electrolyte was recirculated to prevent temperature gradients upon operation, as shown in Figure 1f. The four-electrode configuration allowed for:

1. In operando monitoring of cathode overpotential, anode overpotential, and cell voltage.
2. Simultaneous in situ electrochemical impedance spectroscopy of the cathode, anode, and full-cell.
3. Half-cell and full-cell distribution of relaxation times (DRT) analysis.

This configuration is an evolved version of our previous work.^32^

#### 2.3.3 Dual Reference Electrode Measurement Protocol

Dual reference electrode measurement is schematically shown in Figure 2. The assembled MEA was subjected to a preconditioning chronopotentiometric operation at 1.0 A cm^–2^ at 20 °C for 60 min with 1.0 M KOH solution as a supporting electrolyte. The preconditioning operation reduces the effect of inhomogeneities arising from diffusion-controlled processes and the equilibration of the MEA with its new surroundings. LSV was performed at a scan rate of 25 mV s^–1^, and the anode and cathode overpotentials were calculated based on the measured half-cell potentials using eqs (S10) and (S11), respectively.

**Figure 2:**
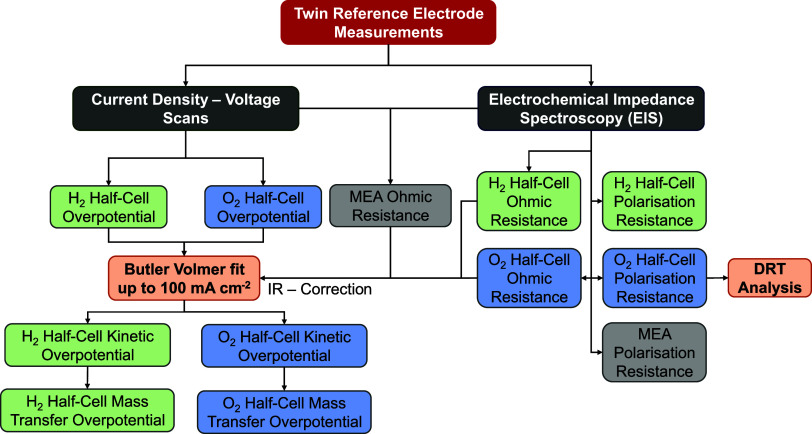
Methodology used to isolate and decouple half-cell and membrane contributions using multiple reference electrodes.

Consequently, EIS scans were performed in pseudogalvanostatic mode with biases 0.1, 0.3, 0.5, 0.7, and 1.0 A cm^–2^ and 5.0 mV amplitude. The EIS spectra between the cathode-RHE, complete cell, and anode-RHE were recorded simultaneously, in parallel, using a Zahner Zennium X with 8-channel PAD4 card.

The parallel EIS measurements facilitated the identification of half-cell and MEA series (ohmic) and reaction resistances (charge transfer and polarization). The *iR* correction of the overpotentials was performed using high-frequency resistance EIS data acquired at 0.1 A cm^–2^. The *iR*-free overpotentials were fitted to the Butler–Volmer characteristics up to 100 mA cm^–2^ and subsequently extrapolated toward the maximum current density of the LSV scan. The discrepancy between the *iR*-free overpotential and the extrapolated Butler–Volmer overpotential was attributed to the mass transfer overpotential of the respective half-cell.

Additionally, EIS data quality was validated through the Kramers-Krönig (KK) residual test (±1.0%) before being processed for distribution of relaxation times (DRT) analysis of both the half-cell and MEA. The DRT approach enables the decoupling of electrode processes, distinguishing them from MEA characteristics while identifying possible process overlaps. The methodology is currently being adapted from previous high-temperature solid oxide cell studies, and readers are encouraged to refer to relevant literature for further insights.^17,33–35^ A fixed regularization parameter of λ = 0.15 was used, as determined by the L-curve method.^36^ Only data with a sum of squared residuals below 10^–3^ were considered for further analysis.

### 2.4 Long-Term Degradation Analysis

The commercial MEA with an active area of 25 cm^2^ was evaluated at 1.0 A cm^–2^ for over 1000 h, using a dry cathode with a 1.0 M KOH supporting electrolyte at 60 °C on only the anode, as shown schematically in Figure S1.^37^ Electrolyte circulation was facilitated using a two-channel Golander BT100s variable-speed peristaltic pump, with a pulse damper positioned in series. Flow rate monitoring was conducted using a Bükert Type 8756 Coriolis mass flow meter (MFM), as illustrated in Figure 3. The flow rate was maintained at 45 ± 2 mL min^–1^, corresponding to a liquid distribution of 1.8 ± 0.08 mL min^–1^ cm^–2^ at the O_2_ electrode. This average flow rate distribution prevents catalyst leaching from the PTL.^38^

**Figure 3:**
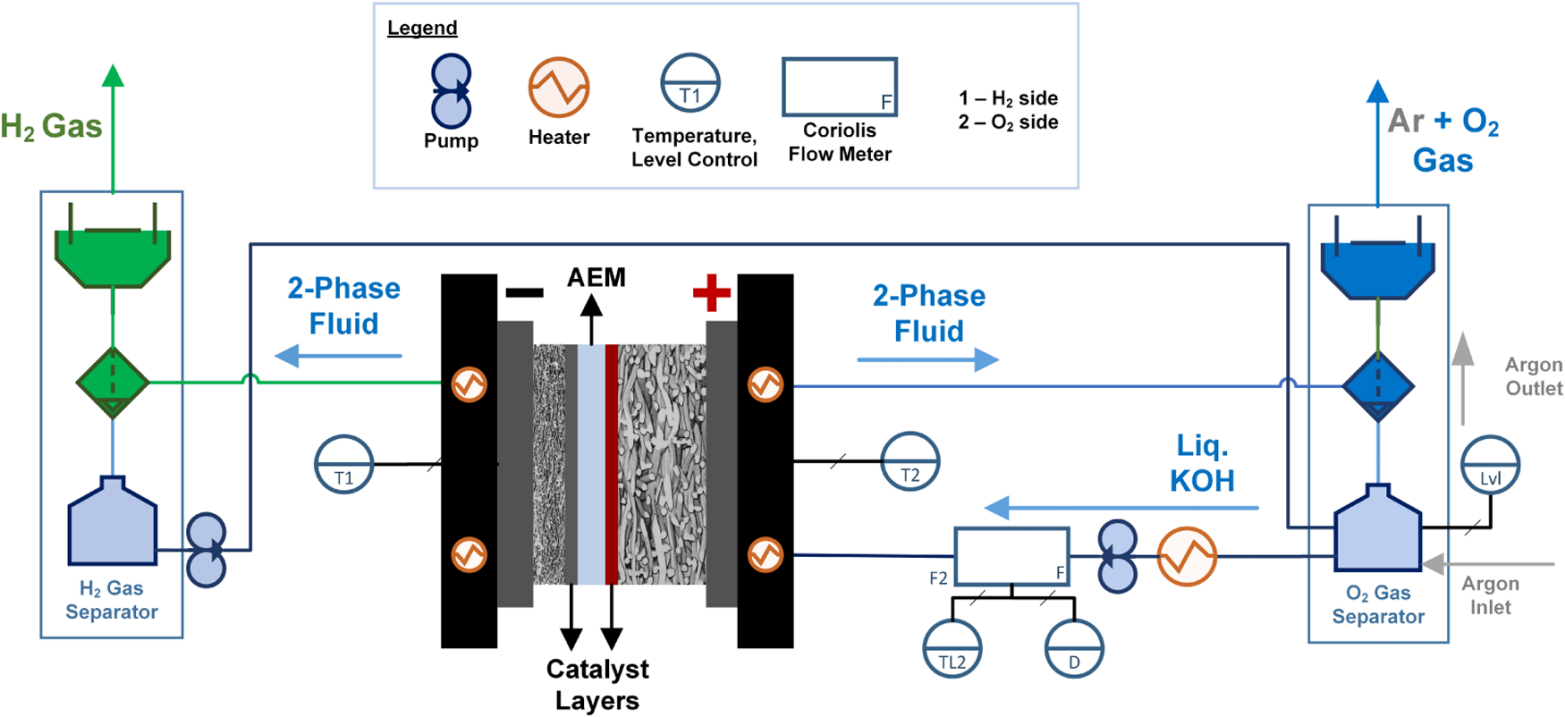
Schematic of the AEMWE setup used for testing long-term durability exceeding 1000 h.

The MFM measured mass flow rate (kg min^–1^), liquid temperature (°C), and liquid density (kg m^–3^). Digital capacitive level sensors were employed at the aqueous KOH reservoir tank to ensure a constant electrolyte level. A reservoir volume of 5 L was utilized. The selection of refill liquid, either 0.1 M KOH or deionized water, was determined based on measured density variations. Argon gas was periodically bubbled through the system every 100 h to displace any dissolved CO_2_. Nitrogen could also be used as an alternative.

Water migrates to the hydrogen electrode through back diffusion and capillary transport driven by membrane hydration, and the HER facilitates local water generation as a function of the applied electric current. This accumulated water separated on the H_2_ electrode side was pumped back to the O_2_ side reservoir to maintain pH balance and minimize excessive refill requirements. The separated H_2_ and O_2_ gases are channeled out through the fume hood.

The in-house built cell housing, constructed from nickel monopolar plates featuring a 3-channel serpentine flow field, was used in isolation with stainless steel 316 L end plates. Heater cartridges are embedded within the end plates, and the cell temperature is regulated by an in-house-created PID control system.

Each end plate contains two heating cartridges, which operate in conjunction with a Type K thermocouple to monitor temperature variations. Additionally, the liquid was slightly preheated to reduce dependency on the cartridge heaters. The PID control system compensates for the temperature rise induced by Joule heating under constant current operation. The whole system was tested for leaks using forming gas (95% N_2_–5% H_2_) before operation.

The MEA was activated at room temperature following a dedicated protocol created by TUB^39^ (see Figure S21 in ref 39) and adapted for AEMWE, as shown in Figure 4. The protocol consists of a series of potentiostatic EIS (PEIS), chronopotentiometry (CP), and chronoamperometric (CA) measurements, divided into conditioning and activity measurement steps. The total duration of the protocol is 16 h, with time dedicated to stability for EIS measurements.

**Figure 4:**
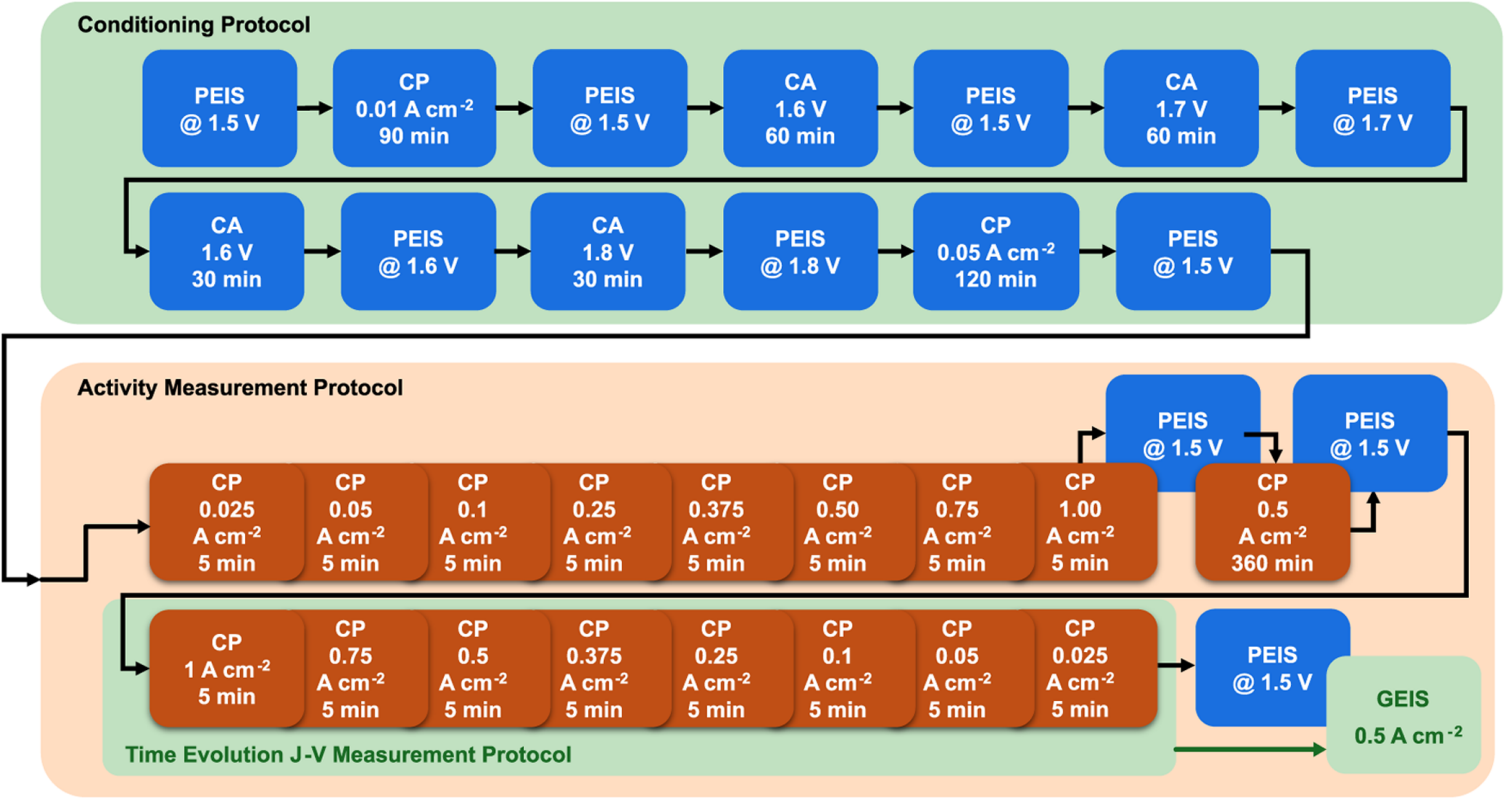
Schematic representation of the protocol used for MEA activation at room temperature and the measurement of *J–V* and EIS characteristics during the long-term durability test.

Upon completing the aforementioned protocol in Figure 4, the temperature was increased to 60 °C. The *J–V* curves for time evolution measurements were plotted using the average current density registered during the last 10 s of the 5 min CP measurements during the backward step scan, i.e., the voltage was evaluated while gradually decreasing the current bias. The EIS at 60 °C was conducted at 0.5 A cm^–2^, with 10% amplitude, at regular time intervals.

For the first 300 h, *J–V* and EIS measurements were performed every 50 h, while between 300 and 1000 h, measurements were taken at intervals of 100 h. Beyond 1000 h, steady-state operation was maintained until complete membrane failure.

Further, the EIS data was assessed for quality using KK residual tests (±1%) and processed to analyze the DRT spectra, thus showing how the impedance measured with dual reference electrodes evolves with time. Although initial measurements were conducted at room temperature, the impedance data is still easily comparable to the data gathered at 60 °C, as all processes will be affected by an increase in frequency position and a decline in magnitude. These two changes originate in the accelerated kinetics and improved electrical contact associated with increased temperature.

## 3 Results and Discussion

### 3.1 Three-Electrode Measurements

Prior to electrochemical testing, a thorough materials analysis was conducted on the commercial catalyst-coated electrodes; see the Supporting Information. The morphology and bulk structure were ascertained through SEM and XTM, providing valuable insight into surface geometry, effective diffusivity, and conductivity. The bulk was probed through XRD and EDX to quantify bulk structure and composition. Surface conditions were evaluated with XPS, thus showing changes in elemental composition from bulk to surface.

Three-electrode measurements were conducted to accurately determine the electrochemical properties of each of these electrodes separately. This enables the differentiation between the anode and cathode performance when both are present in a full cell, as specified in Section 2.3.1. These results are depicted in Figure 5. The Raney nickel cathode required an overpotential of 482 mV to attain 100 mA cm^–2^, as shown in Figure 5a. Similar overpotentials were required by the NiFeO_x_ anode. As shown in Figure 5b, the double-layer *C–V* scans revealed similar ECSAs of 51.5 and 50.75 cm^2^ for anode and cathode, respectively. The ECM used to model the OER and HER EIS spectra is presented in Figure 5c, and it fits the experimental data with less than 3% error for both OER and HER, respectively, as shown in Figure 5d,e. The Nyquist plots are shown in Figure S10a,b. Implementing DRT in the 3-electrode configuration highlights trends similar to the imaginary impedance distribution, as shown in Figure S10c,d.

**Figure 5:**
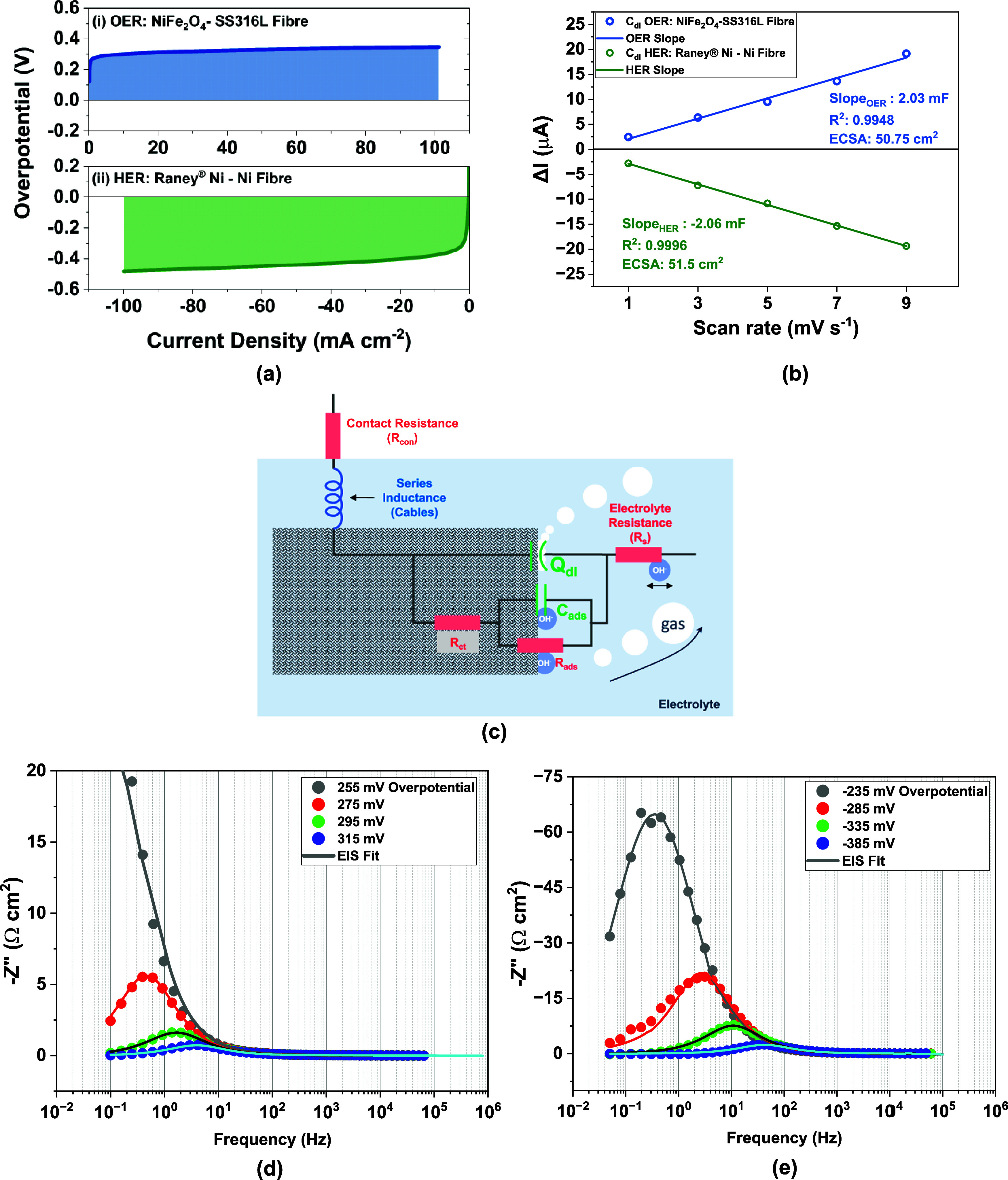
Three-electrode results from the cathode and anode electrodes, showing the (a) linear sweep voltammetry curves and (b) electrochemically active surface area (ECSA) determined based of cyclic voltammetry in the double-layer region. (c) Schematic equivalent circuit model used to determine charge transfer resistance. (d, e) Experimental and modeled electrochemical impedance spectroscopy (EIS) of the OER and HER electrodes, respectively.

Sluggish anodic performances are a familiar sight in water electrolysis as a whole, where the cathode by comparison may easily be catalyzed and typically requires a considerably lower overpotential to reach the same current densities as oxygen evolving anode. Here, the similar overpotentials required to reach 100 mA cm^–2^ for both electrodes clearly indicate the low cathodic performance of the Raney nickel electrode. Polarized EIS spectra in Figure 5d,e show the same trends, where the charge transfer resistance of the cathode at −335 mV is considerably larger (×1.56) than that of the anode at 315 mV.

However, the EIS modeling results in Tables 1 and 2 show that the low-frequency adsorption resistance is clearly limiting in comparison to the charge transfer resistance at these overpotentials for both electrodes (anode 93% and cathode 99%). This indicates that both electrodes struggle with adsorption/diffusion-related phenomena under pseudo-steady-state conditions.^40,41^ XTM shows that both electrodes have similar effective diffusivity, and neither EIS spectra show any clear influence of typical diffusion behavior; thus, the main issues are highly likely related to adsorption. Moreover, the consistent difference in the XTM-determined surface contact area is also seen in EIS spectra, as the HER electrode displays a lower series resistance compared to its anodic counterpart.

**Table 1:**
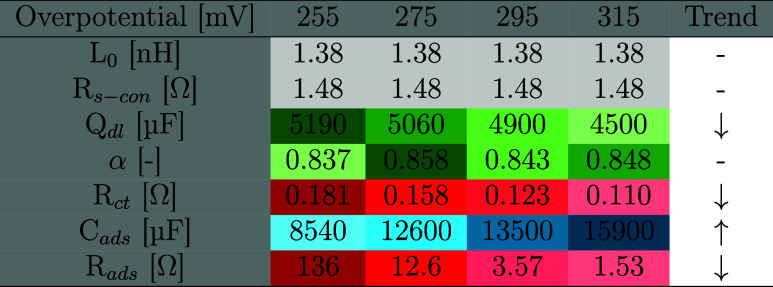
Equivalent Circuit Parameters for OER in the Three-Electrode Configuration

**Table 2:**
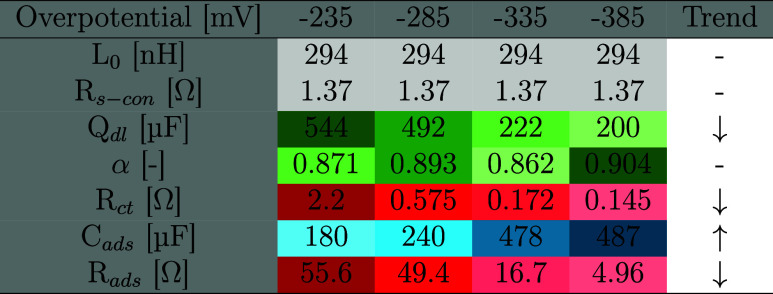
Equivalent Circuit Parameters for HER in the Three-Electrode Configuration

While there are often large disparities between the ex situ ECSA determined through *C–V*s and in situ ECSA determined through polarized EIS,^29,42^ here both *C–V-* and EIS-determined ECSAs show order-of-magnitude similarities. However, both electrodes display a decline in ECSA as the overpotential increases, showing the effective number of available active sites declines due to adsorption/desorption kinetics, which cannot keep the pace set by the rate of electron transfer.

### 3.2 Dual Reference MEA Measurements

#### 3.2.1 Current Density–Voltage Scans

The LSV curves from the entire cell shown in Figure 6a unveil the performance of the MEA in its entirety, which reached 1.0 A cm^–2^ at 2.2 V under ambient conditions. Advanced instrumentation detailed in the Experimental Section allowed a complete decoupling of the cell voltage to show the individual contributions from the anode, cathode, and membrane. Compared to the three-electrode measurements, there is a qualitatively similar trend in the evolution of anodic/cathodic currents, thereby yielding supporting evidence to the lackluster cathodic performance. This is explicitly shown in Figure 6b, where the cathode contributes the majority of the polarization impedance.

**Figure 6:**
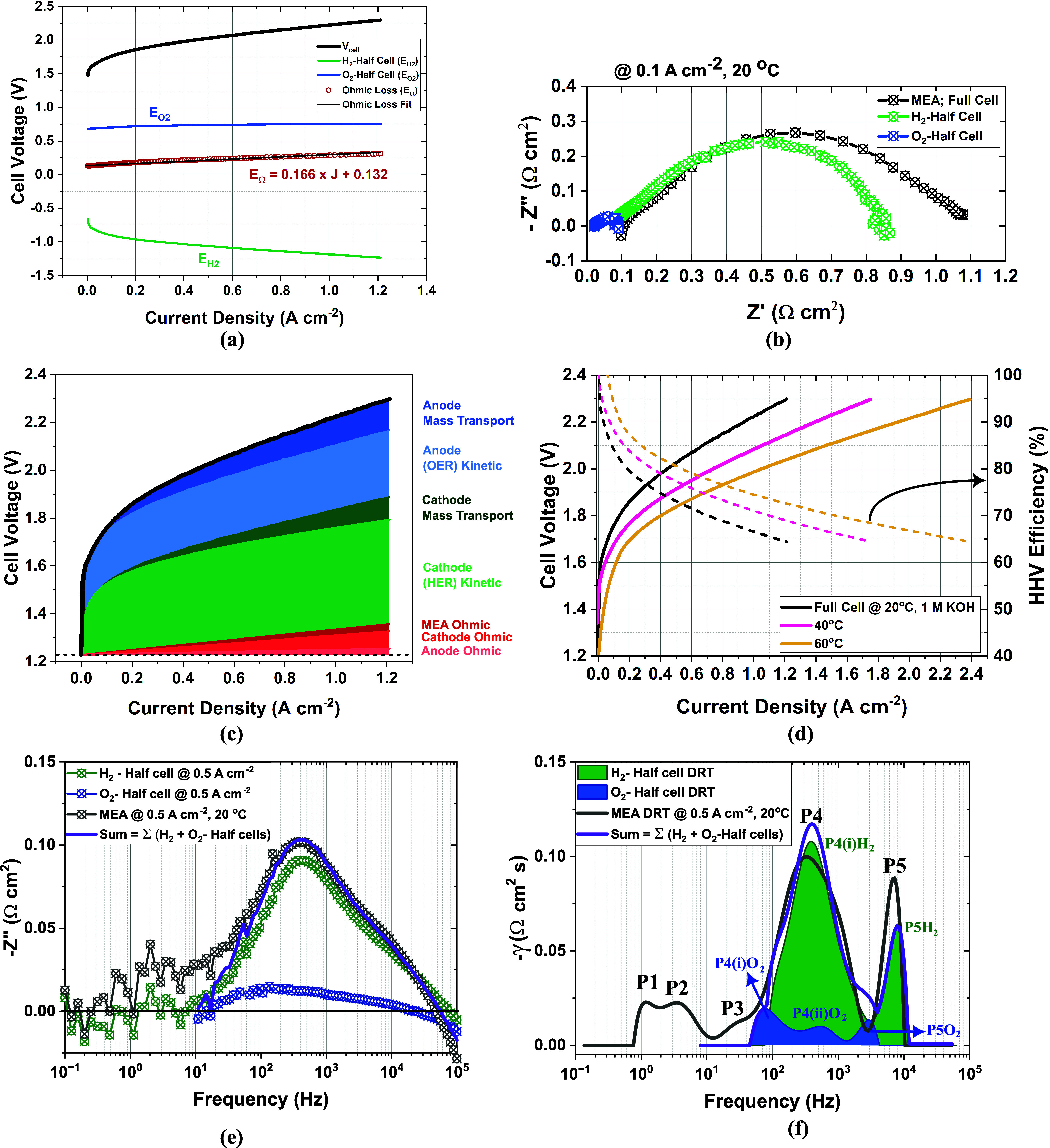
Current density–voltage characteristics of the nickel fiber on Raney nickel ∥X37–50RT∥ NiFeO_x_ on SS316L fiber MEA system with (a) separated anodic, cathodic, and ohmic losses due to dual reference electrode measurements, at 20 °C. (b) The corresponding EIS measurement conducted at 0.1 A cm^–2^, 20 °C allowing the decoupling of Ohmic and polarization resistance contributions. (c) Decoupling of electrode overpotentials, mass transfer, and ohmic losses at 20 °C. (d) Performance of the MEA at 20, 40, and 60 °C and the corresponding HHV efficiency trend as a function of temperature. (e) Frequency distribution of the imaginary impedance component of the MEA and the H_2_–O_2_ half-cell measurements at 0.5 A cm^–2^ (*V*_cell_ > 2.0 V). The corresponding superposition of DRT in (f), based on the smoothened EIS data.

A complete decoupling of the full-cell potential was attained by fitting the Butler–Volmer equations up to 100 mA cm^–2^ to the *iR*-free data yielding the activation overpotential. Pure ohmic contributions were determined through the high-frequency resistance in the parallel EIS measurements, as seen in Figure 6b. The difference between the kinetic overpotentials, ohmic potential loss, and the reversible potential, with the measured value, was determined to be related to the mass transport, as shown in Figure 6a.

The AEM performance improved with increasing temperature, reaching 1.1 A cm^–2^ at 2.0 V at 60 °C, as shown in Figure 6d. The corresponding efficiency of 74.5% HHV was attained at 1.0 A cm^–2^. A maximum current density of 2.4 A cm^–2^ was achieved at 2.3 V.

#### 3.2.2 Electrochemical Impedance Spectroscopy

The parallel EIS measurements enabled the identification of half-cell characteristics at high current densities. However, the frequency range where the sum of the decoupled performances (H_2_+O_2_) matches the MEA performance must be ascertained, as the latter will add minor high-frequency contributions (series resistance, inductance) and more notable low-frequency behavior (OH– diffusion through the membrane). This was exemplified by deviations above 30 kHz, primarily attributed to measurement wire inductance, and membrane-related differences noted at frequencies below 40 Hz.

Decoupled data was thoroughly scrutinized through a statistical analysis of H_2_+O_2_ to MEA similarities to prove a causal relationship to the full-cell data, thereby validating the methodology. Residual analysis, performed as the difference between half-cell superposition and MEA performance, indicated statistically acceptable data (±2 standard deviation) within the frequency range of 29–100 kHz, as shown in Figure S11a.

The residual histogram was fitted with a normal distribution and kernel smoothening,^43^ as depicted in Figure S11b. The normal distribution captures the symmetry of the residuals, indicating similarities between half-cell and MEA measurements. In contrast, kernel smoothing is a flexible technique used to capture distribution patterns without relying on specific assumptions. The observed deviations, particularly at the tails and peak, suggest departures from symmetrical normal distributions (Gaussian). This indicates that the reference electrodes may not fully capture the nonlinearities or additional processes associated with MEA measurements.

Nevertheless, the cumulative residuals and root-mean-square deviation (RMSD) plots (Figure S11c,d) demonstrate reasonable comparability between reference electrode–half-cell measurements and those of the MEA. The EIS evolution as a function of current densities for MEA, O_2_ half-cell, and H_2_ half-cell data is shown in Figures S12a,b and S13a–d.

The successful decoupling of the anode and cathode impedance is further validated by comparing them to the intrinsic three-electrode performance in Figure 5. Clear similarities arise when comparing the decoupled full-cell performance to that measured in a three-electrode setup. The magnitude of both cathodic impedances far exceeds the anodes. Moreover, the frequency distribution reveals the same large cathodic polarization resistance at higher frequencies than noted for the anode. These qualitative similarities emphasize the successful decoupling of the full-cell performance.

The imaginary impedance in Figure 6e revealed that the MEA performance at 0.5 A cm^–2^ was largely determined by the cathode. Specifically, high-frequency (*f* >2000 Hz) processes of the full cell were largely due to the cathode, as was the middle-frequency (50 Hz < *f* < 2000 Hz) polarization resistance. This region shows single dominating peaks at 100, 400, and 400 Hz for H_2_, O_2_, and MEA, respectively. Moreover, these are all obvious composite peaks, as evidenced by clear shoulders on both sides of their maxima.

Low-frequency (*f* < 50 Hz) behavior is also ascribed to the cathode. This is likely affected by the slightly lower pore volume of the cathode, as shown by the tomography in Figure S4j. Examining these trends over several current densities in Figures S13a,b, we find the same trends persist. One noteworthy aspect is the degree to which the cathode impedance improves while the anode remains comparatively similar regardless of current density. It is quite likely that an efficient cathode would surpass the performance of the anode, leading to current density dependency on which reaction is rate-limiting. Cathodic contributions would dominate under low currents, while the familiar anodic contributions would dominate under high currents, i.e., industrial conditions.

To delve deeper into the composite impedance peaks in Figure 6e, the corresponding DRT was determined as exhibited in Figures 6f, S12 and S13e,f. Figure 6f illustrates half-cell performances alongside the MEA, exhibiting good overlap within the statistically determined frequency range. Three distinct processes were identified for both the H_2_ and O_2_ half-cell.

The cathode DRT at 0.10 A cm^–2^ (Figure S13e) shows a large composite peak with 4 clear peaks between 10 and 4000 Hz, with a separate high-frequency peak around 10,000 Hz. Increasing the current bias affects both the frequency and magnitude for all 4 contributions within the composite, while the high-frequency process remains unaffected in these aspects. Moreover, given the shape change of the composite peak, it is clear that its contributors have different sensitivities to current bias.

The main contributor (P4­(i)­H_2_) is the most sensitive, given by its decline in magnitude and increment in frequency, where the latter aspect has resulted in the masking of contribution P4­(ii)­H_2_ and P4­(iii)­H_2_. Finally, contribution P3H_2_ follows a similar change as P4­(i)­H_2_, though to a lesser degree, resulting in its greater visibility as contribution P4­(i)­H_2_ moves away through increasing its frequency position. Additionally, P2H_2_ vanishes with increasing current densities. These trends heavily imply that contributions P4­(i)­H_2_-P4­(iii)­H_2_ are related to HER kinetics, while P5H_2_ is OH^-^ ion transport at the HER electrode..

While a one-to-one comparison of DRT peaks between different systems is unrealistic, there are several comparable nickel-based HER materials in the literature^20,44,45^ which exhibit similar peak-bias dependencies within similar frequency ranges. However, peaks similar to the bias-independent P5 are labeled as instrumentation-related,^20^ which is clearly not the case here.

A high-frequency position around 10,000 Hz would plainly indicate that it is affiliated with a fast electron charge transfer process, only slightly slower than the series resistance (∼50,000 Hz). Given the somewhat greater presence of Nafion resin on cathode as determined by XPS analysis, it is possible that this peak arises from its associated electrically insulating properties.

The O_2_-cell displayed three fairly well-resolved peaks, all at moderate frequencies. Conversely to the cathode, the anode DRT was remarkably similar for all degrees of current density bias (Figure S13f). The three well-resolved peaks all shifted homogeneously to higher frequencies upon increased polarization, while dropping slightly in magnitude. Given their synchronous relationship to each other, these peaks likely originate in OER kinetics. Similar peaks were noted for a comparable spinel OER catalyst, though the frequency position was somewhat lower, which is likely related to the low current density employed during the impedance measurement,^46^ likewise so for a recent publication on NiOOH anodes, where three characteristic peaks were determined at lower frequencies.^44^

Generally, most intermediate-frequency processes are associated with charge transfers in the MEA. In an HER system employing a Raney nickel electrocatalyst, the charge transfer resistance is significantly higher than the optimized NiFeO_*x*_ OER counterpart. The high-frequency ionic transport process in the catalyst layer suggests that the Volmer–Heyrovský reaction for HER is the dominant contributing factor.^47^

Going further down in frequency, the MEA itself displayed clear, causal peaks in the low-frequency range. Three low-frequency processes were identified in the DRT from the MEA, including a doublet at 1.5 and 3.6 Hz, along with a slightly higher-frequency process at 25 Hz. Any peaks below 100 Hz are associated with HER and OER transport processes, such as bubble formation.^47,48^ To this effect, positioning the reference electrodes is key to avoid issues where evolving bubbles interfere with the aqueous KOH medium between the PTL and the RHEs, such that frequencies beyond the typical low-frequency range are affected.

The presence of three distinct peaks in two separate frequency ranges, 1–10 Hz (P1 and P2) and 10–100 Hz (P3), may be attributed to different stages in bubble development but also variations in slower hydrogen and oxygen electrode kinetics in the 100 Hz range. Bubble growth typically progresses through four stages.^49,50^ In the first stage, the electrolyte surrounding the reaction site becomes supersaturated, initiating bubble nucleation (with a bubble radius of *r* = 0). In the second stage, the bubble expands spherically until it reaches the pore size of the porous transport layer (PTL). Upon reaching pore size, the third stage begins, where the bubble adopts a cylindrical form and continues to grow along the pore length until it spans the full PTL thickness. In the final stage, the bubble exceeds the PTL thickness and enters the adjacent flow channel.

Bubble-related impedance contributions predominantly occur during the second and fourth stages. The second stage, emerging from the nucleation-driven growth, is generally reflected in the 10–50 Hz frequency range, while the fourth stage, driven by buoyancy and drag forces, corresponds to lower frequencies between 0.03 and 1 Hz.^50^ In the presence of 1 M KOH, such bubble-induced impedance features and their characteristic frequencies are expected to shift compared to proton exchange membrane water electrolysis (PEMWE). Therefore, the adaptation of PEMWE-based analytical techniques to anion exchange membrane water electrolysis is especially relevant—particularly at the anode, where oxygen evolution and associated bubble dynamics are significantly influenced by the alkaline environment.^51^

Based on the DRT analysis (Figure 6f), it is hypothesized that P1 and P2 correspond to buoyancy- and drag-driven bubble behavior. Specifically, P1 is attributed to oxygen bubbles and P2 to hydrogen bubbles, since oxygen bubbles are typically larger and less mobile, resulting in a lower characteristic frequency. Meanwhile, P3 likely corresponds to the nucleation of both gases, assuming simultaneous initiation at the respective electrode interfaces.

Furthermore, the peak at 25 Hz could be systematically linked to instrumentation effects;^24^ however, this remains unclear in Supporting Information Figure S12a–d, as the peak varies with applied current density. The low-frequency MEA peaks shift toward lower magnitudes and faster frequencies, demonstrating a clear impact of the applied current density.

The applied current density increases the frequency position of P4­(i)­H_2_, while P5H_2_ remains constant, as shown in Supporting Information Figure S12e. The P4­(i)­O_2_ and P4­(ii)­O_2_ processes, representing the OER charge transfer, also decrease with increasing current density, while P5O_2_ remains constant, as depicted in Figure S21d. The processes identified by DRT are summarized in Table 3.

**Table 3:** DRT Peaks Association with Processes Based on Experimental Observations and Literature^24^

**MEA peaks**	**H**_2_ **half-cell peaks**	**O**_2_ **half-cell peaks**	**frequency range (Hz)**	**process description**	**refs**
P1			1–5	water and gas diffusion-related process of HER, OER electrodes	this work extending^24^
P2			5–20		
P3			25–80		
P4	P4(i)H_2_	P4(i)O_2_,	60–2000	HER, OER charge transfer	^24^
		P4(ii)O_2_			
P5		P5O_2_	2200–3200	OER ion transport	this work extending^24^
P5	P5H_2_		7000–9000	HER ion transport	this work extending^24^

A deeper understanding of the three low-frequency peaks and their correlation with OER and HER requires a material sensitivity analysis combined with electrolyte feed configuration, which will be explored in future studies. Nevertheless, the current study successfully decouples the physical bubble-related process from electrochemical processes through the implementation of DRT.

### 3.3 Large-Area Single Repeating Unit Measurement

Deciphering the steady-state, pristine performance provided valuable information on the initial state of the cell; however, the evolution of this performance over time under industrial operating conditions remains elusive. To shed light on the matter, the cell area was increased from 1 to 25 cm^2^, the temperature increased to 60 °C, and the cathode KOH-feed was removed. Changes in impedance, DRT, and steady-state polarization behavior were evaluated over 1000 h of operation, thus providing invaluable information on the stability and efficacy of the employed materials.

The steady-state chronopotentiometric dry cathode feed operation of nickel mesh-Raney nickel ∥X37–50RT∥ NiFeO_*x*_-SS316L fiber exhibited progressively increasing performance throughout the experiment, as shown in Figure 7a. A well-designed control system kept the intermediate-scale cell at its rated values, where the cell temperature remained stable within ±0.5 °C during steady-state operation and fluctuated by ±5 °C when the applied current was interrupted for measurements. The PID-based temperature controller effectively compensated for the exothermic behavior of the cell during steady-state operation. Upon stopping the bias, a temporary heat loss caused an initial temperature drop, triggering the PID controller to compensate, resulting in an overshoot, as observed in Figure 7b. Electrochemical measurements were initiated only after the system stabilized at 60 ± 0.5 °C.

**Figure 7:**
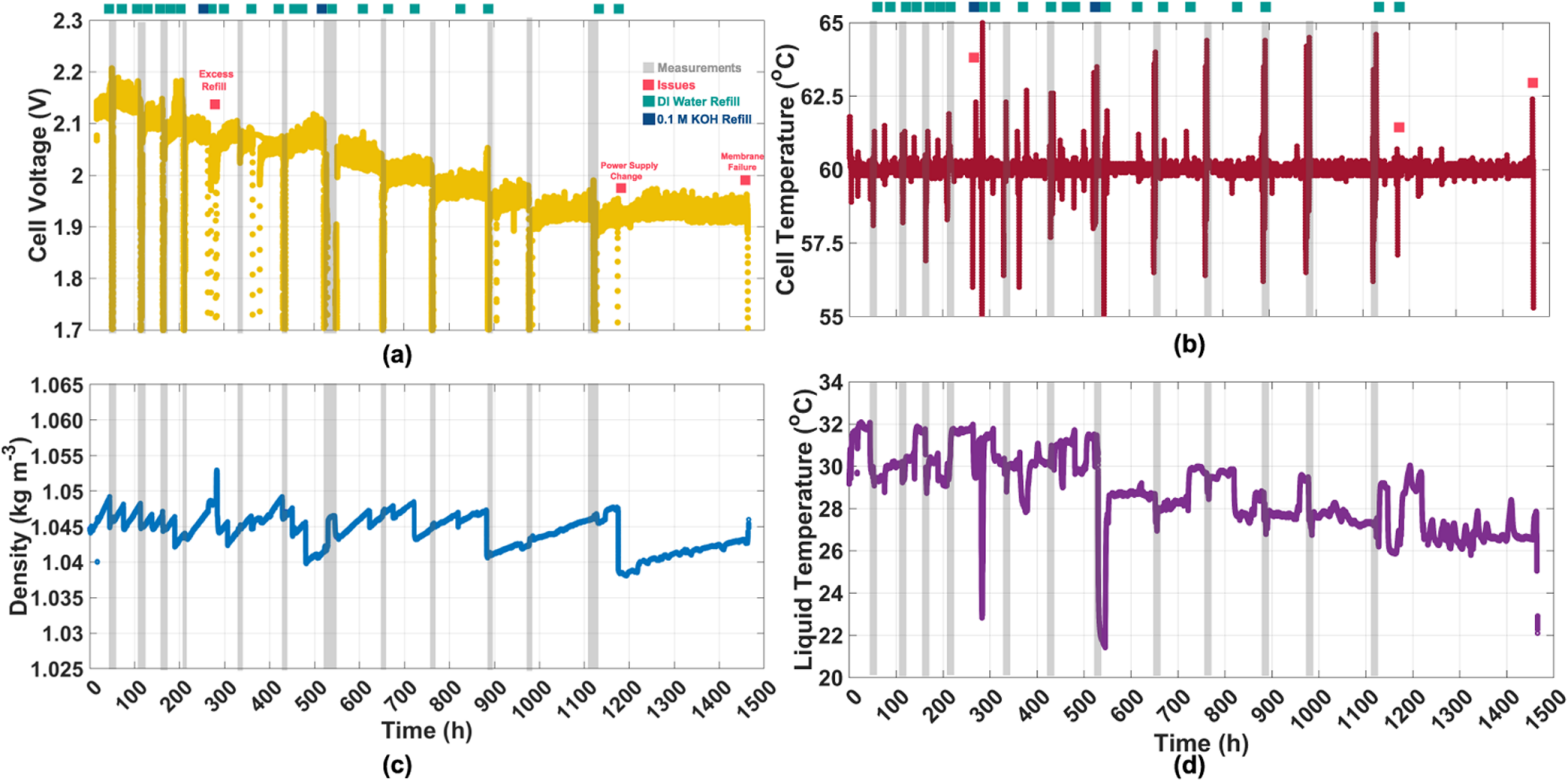
(a) Time evolution of the cell voltage in 1.0 M KOH, at 60 °C, 1.0 A cm^–2^, with indicated 0.1 M KOH/D.I. water refill points. (b) Temperature stability between 60 ± 2.5 °C during measurements/refill and 60 ± 0.5 °C during steady-state operation. (c) Density of KOH electrolyte changing due to consumption/refill and (d) the corresponding liquid temperature at the mass flow meter, before inlet. The plotted data have not been filtered for noise or any excessive peak values.

The solution density was maintained within 1.04–1.05 kg m^–3^ by periodically refilling with deionized water or 0.1 M KOH to compensate for KOH loss caused by evaporation or saline mist formation from the oxygen evolution reaction. The fluctuation in solution density remained below ±0.8%, as shown in Figure S14.

This density range ensured a stable 1.0 M KOH concentration, with variations found to be negligible, as depicted in Figure 7c. After 1000 h, the refill frequency was reduced to fewer than three times to evaluate its impact on cell performance. No significant effect was observed, based on the constant cell voltage.

Furthermore, the liquid temperature, which directly influenced the measured density, remained stable during electrochemical measurements, as shown in Figure 7d. Refill quantities affected the liquid temperature and, consequently, the density, necessitating a more frequent automated adjustment process.

A gradual increase in the steady-state *J–V* performance was observed throughout the long-term degradation test (Figure 8a), thereby supporting the chronopotentiometry data in Figure 7a. Initially, a clear improvement in performance is registered after the first 50 h, which may be ascribed to the well-known break-in effect. This lowers the series resistance, but in this case also increases the total polarization resistance, as expressed in Figure 8d. DRT in Figure 8e shows that the change in polarization resistance is associated with a peak separation of P3 and P4, implying slower HER kinetics. Simultaneously, the anode polarization resistance should be decreasing through the favorable transformation of α-NiOOH to β-NiOOH,^52^ though this improvement was likely already in place due to the activation protocol (see the Experimental Section) carried out before hour 0

**Figure 8:**
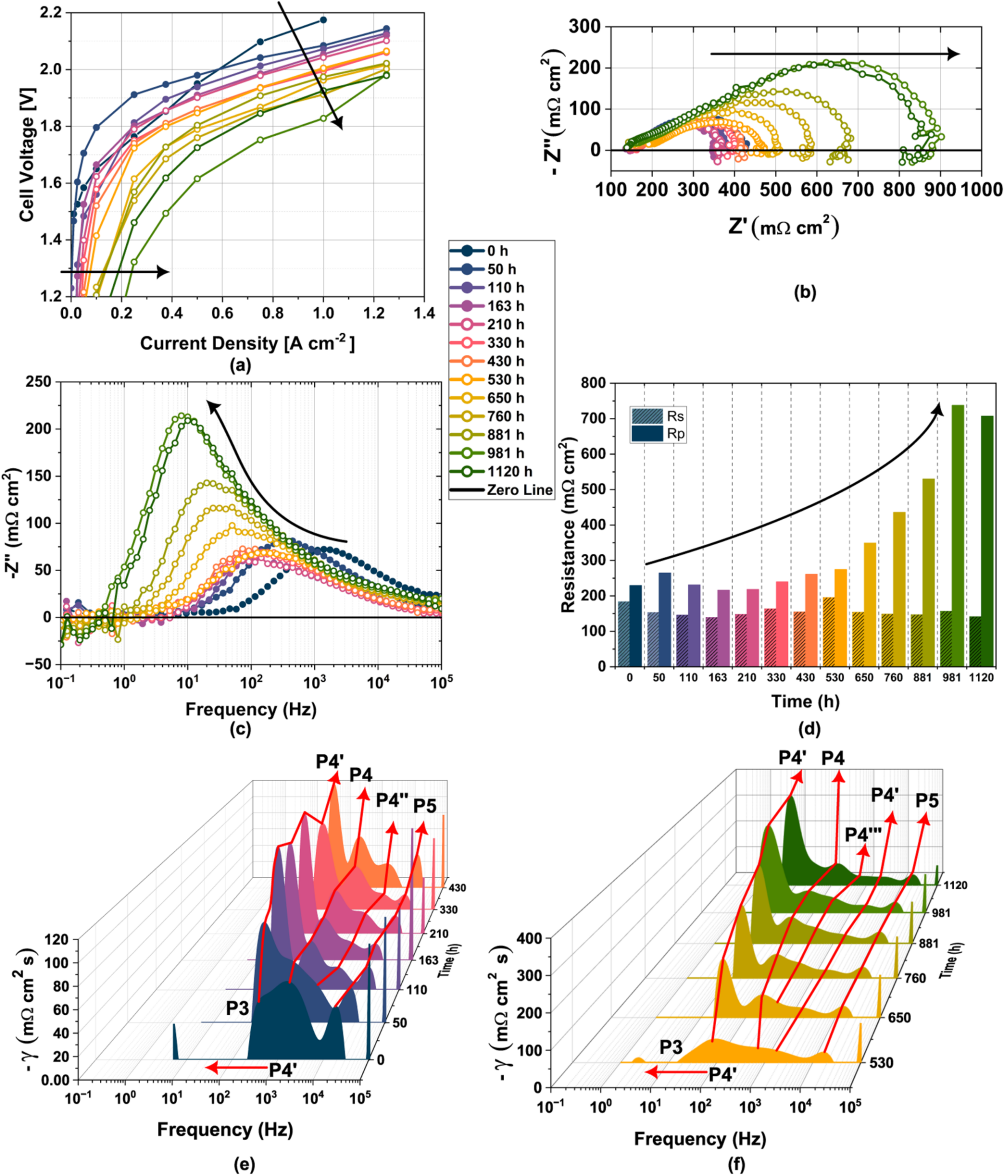
Time evolution of (a) current density–voltage characteristics of Raney nickel on nickel mesh ∥X37–50RT∥ NiFeO*_x_* on SS316L in 1.0 M KOH, at 60 °C (without *iR* correction), (b) the Nyquist characteristics with increasing polarization resistance at 0.5 A cm^–2^, (c) the corresponding Bode plot highlighting slower frequency shift of the MEA charge transfer resistance, (d) series and polarization resistance, (e) distribution of relaxation times (DRT) analysis from 0 to 500 h, and (f) the DRT spectra of the optimized EIS measurements from 500 to 1000 h.

Polarization resistance decreases between 50 and 210 h, thus accentuating the peaks P4, P4′, and P4″, while P3 merges into P4′. This lowers the total impedance to slightly below its initial value as seen in Figure 8b,d, implying stable operation which is also seen in the steady-state *J–V* curves in Figure 8a. Between 210 and 430 h, peaks P4 and P4′ gradually shift toward lower frequencies, signaling degradation.

The onset of leakage currents at 1.2 V became prominent after 530 h, indicating partial membrane failures, likely due to pinhole formation.^53–55^ The origin of these pinholes is complex, but we hypothesize that they ultimately originate from the degradation of the Nafion perfluorinated resin solution. As detailed by the XPS analysis in Figures S6–S8, both anode and cathode electrodes contain a significant amount of Nafion resin solution, which is an efficient binder and frequently employed for, e.g., hot-pressing MEAs. Considering the notable pressure applied from each side of the cell (∼12 kN ≈ 48 bar), it is possible that the MEA was partially hot-pressed together. This would occur only in small regions with temperature hot spots, and would not initially manifest as short circuits due to the electrically insulating nature of Nafion.

However, Nafion is not stable in an alkaline electrolyte,^8,56^ as it is prone to nucleophilic attack, in turn exacerbated by the oxidizing atmosphere of the anode. Upon degradation, these hot-pressed points will leave the underlying AEM either weakened or incomplete, gradually increasing the current leakage. Moreover, the degradation of Nafion perfluorinated resin solution also accounts for increments in the polarization resistance, as its degradation products may strongly adsorb onto active NiO sites and lower local pH.^56^ While stable coadsorption of SO_3,ads_ and OH_ads_ is possible under operational conditions similar to ours, it would still imply the loss of an active site and thereby a decreased rate of oxygen catalysis.

Despite the presence of leakage currents, the cell voltage remained stable under operation. This emphasizes the importance of performing steady-state polarization tests in situ to confirm a operational, safe cell, as it is clear that the chronopotentiometry data alone fails to capture these nuances. This highlights the necessity of measuring hydrogen crossover, *J–V* curves, and water diffusion volume at the cathode (dry cathode feed operations) to ensure accurate long-term durability assessments. For large-scale membrane applications, these factors must be considered.

Pinhole formation is not entirely detectable via electrochemical impedance spectroscopy (EIS) at high current densities (0.5 A cm^–2^) as in this study. However, since the membrane retains its ionic properties after partial failure, further analysis of evolving EIS trends remains valid, with a focus on catalyst layer degradation. The Nyquist plot of the EIS spectra exhibited stable to decreasing polarization resistance between 0 and 210 h, as shown in Figure 8b.

Polarization resistance consists of anodic and cathodic charge transfer resistance, including ionic, electronic, and mass transfer contributions. After 530 h, a significant increase in polarization resistance was observed, accompanied by a reduction in charge transfer frequency, as indicated in the imaginary impedance plot (Figure 8c). The quantified series resistance exhibited a slight decreasing trend, while polarization resistance increased, as shown in Figure 8d.

This changing trend is crucial for implementing the distribution of relaxation time analysis based on Tikhonov regularization. The positive imaginary component of impedance (+Z″, below the zero line) is omitted in the DRT analysis, resulting in a blank region in this range. Frequencies below 1 Hz are significantly affected by bubble formation, which impacts the accuracy of the DRT analysis. Since DRT is primarily used to interpret electrochemical processes, the physical effects of bubbles are disregarded.

The changing EIS measurement conditions, as mentioned in Section 2.3.3, do not affect the DRT spectra, as shown in Figure 8f. The degradation mechanisms of peaks P4 and P4′ are distinctively captured, with P4′ reducing in frequency and visibly increasing in resistance over time, indicating the largest single process contribution to degradation.

Additional peak P4‴ emerges during the 530 to 1120-h degradation test, showing clear deconvolution. These processes are related to the formation zones of P4­(i)­O_2_, P4­(ii)­O_2_, and P4­(i)­H_2_. Furthermore, P5 remains nearly unchanged during this period, although a slight increase was observed between 230 and 530 h.

Further, sensitivity analysis of materials, current density, electrolyte feed type, temperature, and KOH concentration is necessary to enhance the electrochemical understanding of P4′ and P4″. Once these processes are well characterized, an equivalent circuit diagram can be fitted using DRT as a quality criterion, employing a complex nonlinear least-squares fit method.

Combining these analyses with post-test material characterization, such as XPS, Raman spectroscopy, and SEM-EDS, can provide insights into oxidation, leaching, and ionomer decomposition of electrocatalysts, potentially narrowing down the cause of degradation to material functionality.^57–73^

## 4 Conclusions and Future Outlook

This study conducted a comprehensive structural and electrochemical characterization of commercial anion exchange membrane water electrolyzer (AEMWE) components, integrating material diagnostics, electrochemical testing, and advanced analytical techniques to assess performance and degradation mechanisms. Material diagnostics were performed on pristine samples of the Sustainion X37–50 AEM, NiFeO_x_ anode, and Raney nickel cathode to establish their initial structure, composition, and surface properties.

The dual reference electrode setup enabled the decoupling of anode and cathode overpotentials in full-cell operation, facilitating half-cell EIS measurements and isolating individual electrode contributions. Additionally, residual analysis validated half-cell and MEA decomposition, ensuring consistency between individual electrode contributions and full-cell performance. Residual analysis confirmed a strong correlation between individual half-cell resistances and overall MEA performance. The 1 cm^2^ MEA test achieved a peak performance of 1.0 A cm^–2^ at 2.2 V under ambient conditions and 1.1 A cm^–2^ at 2.0 V at 60 °C, corresponding to an HHV efficiency of 74.5% at 1.0 A cm^–2^.

The long-term MEA test, with an active area of 25 cm^2^, was conducted at 1.0 A cm^–2^, 60 °C, in 1.0 M KOH for 1000 h. A significant increase in polarization resistance was observed, primarily due to electrode resistance changes and mass transport limitations. Despite this, MEA performance unexpectedly improved over time, likely due to membrane degradation altering ion transport properties. However, this did not indicate an overall system improvement.

Distribution of relaxation times (DRT) analysis, coupled with dual reference electrode studies, identified key degradation mechanisms. Low-frequency processes (1.5–25 Hz) were linked to bubble formation and mass transport limitations. Intermediate-frequency processes (50–2000 Hz) were associated with charge transfer resistance, while high-frequency processes (>2000 Hz) corresponded to ionic transport resistances. Additionally, double-layer capacitance increased over time, suggesting structural modifications in electrode porosity.

This study highlights the importance of integrating robust DRT methodologies, three-electrode EIS measurements, and dual reference electrode techniques to accurately identify and quantify loss mechanisms in AEMWEs. The combination of material diagnostics and advanced electrochemical testing provided deeper insights into electrode and membrane behavior under long-term operation.

Future studies should focus on membrane durability improvements to mitigate ion transport alterations, catalyst optimization strategies to reduce charge transfer resistances, and electrolyte feed configuration adjustments to enhance overall performance stability. These findings contribute to the development of durable and efficient AEMWE technology, bridging the gap between research and commercial viability.

## Supplementary Material



## Notes

Additional experimental details, materials, and methods such as reference electrode corrections, overpotential calculations, and supporting electrolyte density fluctuations; material characterizations including XRD, SEM-EDX, microtomography, and XPS data; electrochemical characterization including double-layer capacitance, EIS, and DRT; and statistical analysis confirming similarities of dual reference electrode measurements with MEA data (PDF)
