# Supplementary material for: Combinatorial Use of Reference Electrodes and DRT for Disentangling AEM Electrolyzer Losses
Source: Energy Fuels. 2025 Aug 15;39(34):16485–500. doi: 10.1021/acs.energyfuels.5c01799 (PMC12400285; doi:10.1021/acs.energyfuels.5c01799)
Supplement: Supplementary file 1 [file ef5c01799_si_001.pdf]

Supporting Information

Combinatorial Use of Reference Electrodes and DRT for disentangling AEM Electrolyser Losses

Suhas Nuggehalli Sampathkumar[*,a], Thomas Benjamin Ferriday[a,b], Samaneh Daviran[a], Hamza Moussaoui[a], Philippe Aubin[a], Khaled Lawand[a], Mounir Mensi[c], Pascal Alexander Schouwink[c], Albert Taureg[d], Vanja Subotić[e], Arthur Paul Lucien Thévenot[f], Fabio Dionigi[f], Peter Strasser[f], and Jan Van Herle[a]

[a] Group of Energy Materials, École polytechnique fédérale de Lausanne (EPFL), Rue de l'Industrie 17, Sion, 1951 Valais, Switzerland

[b] Centre for Materials Science and Nanotechnology, University of Oslo, Gaustadalléen 21, 0349 Oslo, Norway

[c] X-Ray Diffraction and Surface Analytics Facility, École polytechnique fédérale de Lausanne (EPFL), Rue de l'Industrie 17, Sion, 1951 Valais, Switzerland

[d] ENAC Interdisciplinary Platform for X-ray micro-tomography (PIXE), École polytechnique fédérale de Lausanne (EPFL), Station 18, Lausanne,1015, Vaud, Switzerland

[e] Institute of Thermal Engineering, Graz University of Technology, Inffeldgasse 25/B, Graz, 8010, Austria

[f] Department of Chemistry, Technical University Berlin, Straße des 17, Juni 124, Berlin, 10623, Germany

[*] Corresponding author; E-mail: suhas.nuggehalli@epfl.ch

Experimental methods

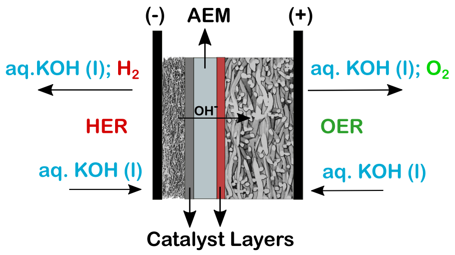

(a)

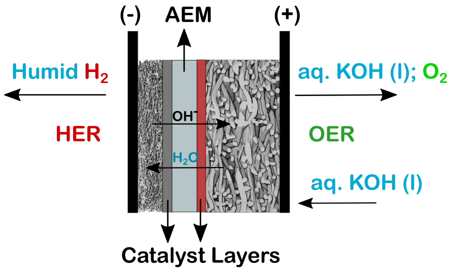

(b)

Figure S1: **(a)** The operating principle of anion exchange membrane water electrolysers in standard wet cathode feed mode and **(b)** dry cathode feed mode.

Reference Electrode Corrections: The overpotentials governing electrode kinetics are calculated based on measurements from the reference electrode. Since the electrolyte in the Ag/AgCl electrode differs from the measurement medium (3.0 M KCl instead of 1.0 M KOH), a pH correction is required to calculate overpotentials with respect to the reversible hydrogen electrode (RHE) rather than the standard hydrogen electrode (SHE). Overpotentials calculated with respect to RHE provide more accurate measurements than those with SHE, as local pH variations are taken into account. Overall, RHE is preferred for practical applications, while SHE is typically favoured in theoretical contexts. Therefore, the first step is to determine the potential of Ag/AgCl reference electrode with respect to RHE, as shown in Eqs. S1 and S2.

$$E^{o}_{Ag/AgCl-SHE} = +\ 0.210\ V \tag{S1}$$

$$E_{Ag/AgCl-RHE} = E^{o}_{Ag/AgCl-SHE} + 2.303 \cdot \left(\frac{RT}{F}\right) \cdot pH \tag{S2}$$

where, $R = 8.314$ kJ mol $^{-1}$ is the universal gas constant, T K is the temperature of the working electrolyte and F $=$ 98453.33 C mol^{-1} is the Faradaic constant.

At pH 14, Eq. S2 simplifies to Eq. S3, which is practically relevant for setting the measurement range in the potentiostat. The applied potential in RHE then changes to Eq. S4.

$$E_{Ag/AgCl-RHE} = 0.210 + (0.059 \cdot 14) = +\ 1.036\ V. \tag{S3}$$

$$E_{applied-RHE} = E_{applied-Ag/AgCl} + E_{Ag/AgCl-RHE}$$
$$E_{applied-RHE} = E_{applied-Ag/AgCl} + 1.036\ V \tag{S4}$$

The linear sweep voltammetery (LSV) was performed for the HER and OER electrodes at a scan rate of 1.0 mV s^{-1}. The LSV range for the HER electrode ($E^{H_2}_{applied-RHE}$) was between -610 mV to -1290 mV while that of OER electrode ($E^{O_2}_{applied-RHE}$) was between 510 mV to 750 mV.

Electrochemical impedance spectroscopy (EIS) measurements were conducted in potentiostatic mode at four voltage levels, each with a 5.0 mV amplitude, to analyse the activation kinetics trend. The frequency scan employed 5 steps per decade in the range of 10 mHz to 66 Hz, and 10 steps per decade above 66 Hz. The number of measuring periods was set to 3 and 20 for these respective frequency windows. This protocol was applied universally across all MEA configurations.

An equivalent circuit model (ECM) was employed to describe the electrode kinetics in the three-electrode configuration, where we employed the standard ECM for a single-electron charge transfer step with adsorption as described by A. Lasia [40] and S.S. Jeon *et* al. [57] . While the number of electron transfer steps in the OER exceeds one, the quantitative validity of fitting an impedance spectrum to an ECM which accounts for this is low due to the tightly clustered time constants. A summary of the measurement conditions for the three-electrode setup is provided in Tab. S1. The details of the ECM are discussed in section 3.1.

Table S1: Three-electrode experimental testing conditions.

Test	LSV ($E_{applied\text{-}Ag/AgCl}$)	EIS ($E_{applied\text{-}Ag/AgCl}$)	
	**Range (mV) @ 1.0 mV s^{-1}**	**Bias (mV)**	**Amplitude (mV)**
**OER electrode**	510 to 750	660; 680; 700; 720	5
**HER electrode**	-610 to -1290	-1060; - 1100; - 1160; -1210	

Overpotential Calculation: Overpotential calculations were performed during the post-test data treatment stage. To calculate the HER and OER overpotentials in the working electrolyte at pH 14, the equilibrium (thermodynamic or reversible) potential must first be determined. This equilibrium potential follows the Nernst equation and is provided for HER and OER in Eqs. S5 and S6. However, these potentials are referenced to SHE.

$$E^{o}_{H_2-SHE} = -0.059 \cdot 14 = -0.826\ V \tag{S5}$$

$$E^{o}_{O_2-SHE} = 1.229 - 0.059 \cdot 14 = +\ 0.403\ V \tag{S6}$$

The RHE compensation of the equilibrium potential values for HER and OER is done based on Eq. S7.

$$E_{RHE} = E^{o}_{SHE} + 0.059 \cdot pH \tag{S7}$$

At pH 14, the equilibrium potentials for HER and OER change to Eqs. S8 and S9.

$$\begin{aligned} E_{H_2-RHE} &= E^{o}_{H_2-SHE} +\ 0.826\ V \\ \boldsymbol{E_{H_2-RHE}} &= -0.826 + 0.826 = \mathbf{0}\ \boldsymbol{V} \end{aligned} \tag{S8}$$

$$\begin{aligned} E_{O_2-RHE} &= E^{o}_{O_2-SHE} +\ 0.826\ V \\ \boldsymbol{E_{O_2-RHE}} &= +\ 0.403 +\ 0.826 = \mathbf{1.229}\ \boldsymbol{V} \end{aligned} \tag{S9}$$

Finally, the overpotentials for HER and OER can be calculation as shown in Eqs. S10 and S11.

$$\begin{aligned} \eta_{H_2} &= E_{applied-RHE} - E_{H_2-RHE} \\ \eta_{H_2} &= E_{applied-RHE} \end{aligned} \tag{S10}$$

$$\begin{aligned} \eta_{O_2} &= E_{applied-RHE} - E_{O_2-RHE} \\ \eta_{O_2} &= E_{applied-RHE} - 1.229 \end{aligned} \tag{S11}$$

Spectroscopic

SEM-EDS and XTM

SEM/EDS and XTM was performed to understand the morphology of the electrodes and quantitatively assess the bulk composition. SEM images in Fig. S4a shows the catalyst-covered nickel fibre PTL, where the it comprises many rounded strands crisscrossing each other in the XY-plane. This configuration creates pores into the depth of the PTL, enabling rapid transport of the two-phase flow. The catalyst comprising several variably sized particles, is fairly evenly distributed over the nickel fibre surface and appears to adhere to the nickel fibre strands. Nafion provides the adhesion, where spray coating a Nafion-heavy ink onto a heated substrate would produce this

effect, as shown in Fig. S4a. The greatest distribution of the catalysts is near the surface, which will significantly enhance catalyst utilisation and reduce interfacial contact resistance in the catalyst layer||AEM interface [58].

The non-quantified EDS elemental mapping for the HER (Fig. S4b–d) reveals a uniform distribution of nickel across the catalyst-coated substrate. Oxide species were also detected, alongside an unexpected element, aluminium. The corresponding quantified EDS scan intensities are presented in the supplementary information (Figs. S2a and S3). The principal cathodic elements identified were nickel, oxygen, and fluorine, along with additional elements attributed to the substrate; however, no significant quantities of cobalt or iron were detected. The substrate's contribution to the EDS distribution map is clearly distinguished, as shown in Fig. S4c.

The SEM image of the catalyst coated SS316L PTL in Fig. S4e portrayed a similar overall morphology to the cathode. The stainless steel hexagonal strands of the anodic substrate also form a crisscrossing pattern, albeit with a significantly thicker strands with 14-26 μm faces, same as our prior work [28, 29]. This produces greater pores in Z-plane, which will aid in dispelling the comparatively large oxygen bubbles relative to those formed on the cathode. Catalyst particles display a good distribution, as shown in Fig. S4a, though with a lower intensity than the cathode.

The anode catalyst distribution map shown in Fig. S4f confirms the presence of catalyst-rich regions, predominantly located on the surface of the substrate, thereby enhancing catalyst utilisation. The presence of iron and nickel within the catalyst layer is evident from the non-quantified EDS spectra of the OER catalyst-coated substrate, Fig. S4f. Differences in catalyst and exposed substrate are illustrated through the chromium oxides in Figs. S4g and h, where the former is shown through the darkened parts of the strands, while green highlights exposed support strands. In contrast to the cathode, the anode EDS confirmed the presence of the expected elements, as shown in the supplementary spectra (Fig. S2b). The SS316L fibre contributed relatively less to the EDS distribution map, as indicated in Fig. S4e.

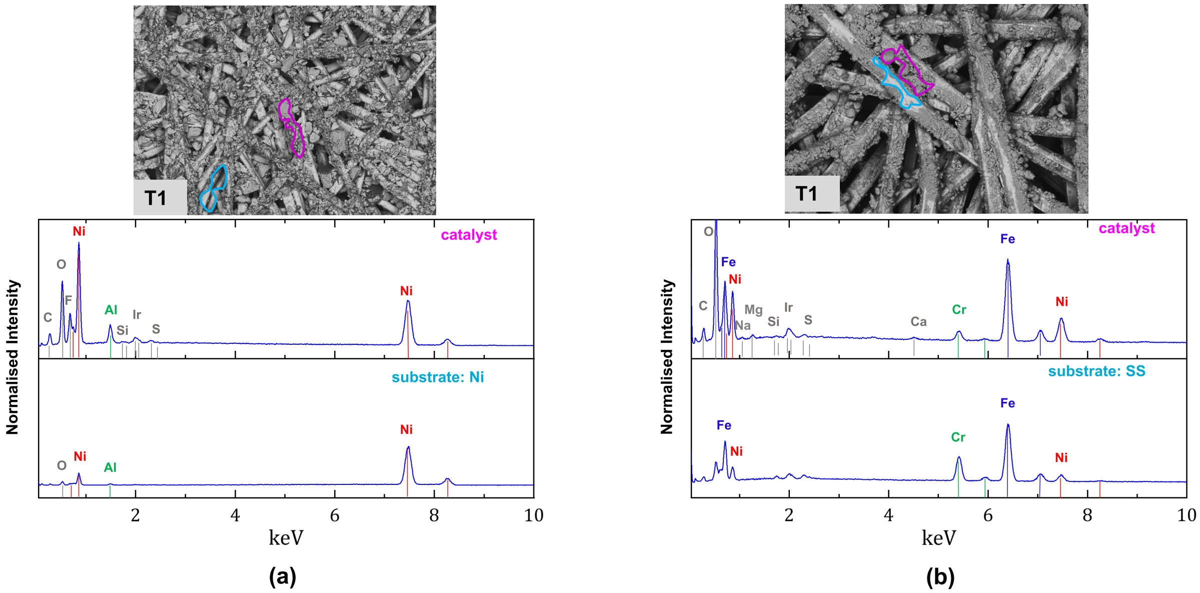

Figure S2: The EDX spectra measured on the deposited particles and the substrate, corresponding to **(a)** HER, Ni-(Fe-Co)-O and **(b)** OER, $NiFeO_x$.

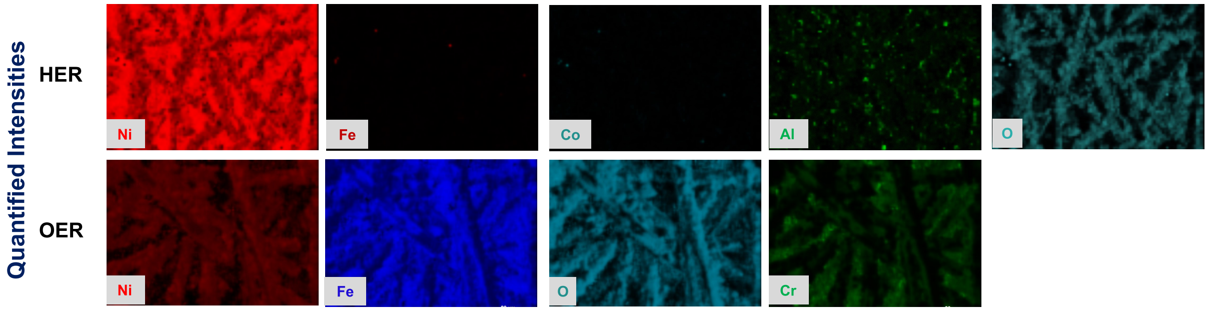

Figure S3: The EDX quantified map measured corresponding to HER, Ni-(Fe-Co)-O and OER, $NiFeO_x$.

The XTM 3D-reconstruction revealed a dense nickel fibre HER substrate matrix with a mean fibre diameter (ϕ_{f-Ni}) of 10 μm as shown in Fig. S4j i) and S4j iii). The mean pore size distribution for nickel fibre (ϕ_{p-Ni}) was calculated to 26 μm, showing a rather low standard deviation. This fits well with the general impression from the SEM picture in Fig. S4a where small, rounded fibres create smaller pores in the Z-plane. The frequency of pore

sizes between 10-45 μm is generally fair, though it declines sharply for pores $\leq$10 μm and $\geq$45 μm. This adheres to the lower pore size limits previously determined for micro-porous layers [59].

The corresponding SSF-OER substrate mean strand diameter (ϕ_{f-SSF}) was more than double, at 22 μm, similar to the SEM-determined strand face-size. Though, the quantity of these strands was naturally lower than that shown for the cathode. The mean pore diameter of the SS316L fibre substrate (ϕ_{p-SSF}) was 45 μm, as represented in Fig. S4j ii) and S4j iv). Moreover, the standard deviation of the SS316L fibre substrate was surprisingly larger. The apparent pore size appeared generally large in the SEM image in Fig. S4e, however this reveals that there is a fair quantity of small and large pores. This distribution of pore size is necessary to adequately handle two-phase flows, such as that typically encountered in electrolyser anodes [59, 60].

Furthermore, the effective diffusivity and conductivity of both the substrates were found to have spacial heterogeneity as summarised in Tab. S2. The reduction in the conductivity was observed to be prominent in the Z-direction i.e. along the thickness of the PTL, between the flow fields and the membrane, by a factor of nearly 10. The reduction is predominately related to the manufacturing process, as the fibres are spun in the XY plane [61]. However, the overall limitation in the conductivity would still likely be attributed to the membrane, due to a lower conductivity of 5-8 orders of magnitude.

Table S2: The anode and cathode PTL substrate properties derived from XTM measurements.

**Substrate**	**Porosity**						**Surface contact area (μm^2/ μm^3)**	**Fiber**					
	**Volume fraction (%)**	**Connectivity (%)**	**Mean diameter (μm)**	**Effective Diffusivity (-)**				**Volume fraction (%)**	**Connectivity (%)**	**Mean diameter (μm)**	**Effective Conductivity (-)**		
Ni Fibre	71.65	100	26.00	x 1.6	y 1.6	z 1.5	8.08 10^{-2}	28.35	100	10.30	x 2.6	y 3	z 11.1
SS316L	67.79	100	44.50	x 1.4	y 1.7	z 1.5	4.62 10^{-2}	32.21	100	22.00	x 1.9	y 3.2	z 7.87

XRD and XPS

X-ray diffraction was performed to quantify crystallographic conditions of the anode and cathode CCS', where as shown in Fig. S5, the majority of the signal originated from the substrates. To this effect, large peaks from the nickel fibre substrate at 44.55°, 51.91°, 76.45°, 92.98° and 98.46° correspond to 111, 200, 220, 311 and 222 planes of metallic nickel respectively [62, 63, 64]. The Rietveld refinement confirmed the cubic Fm-3m (225) structure with a

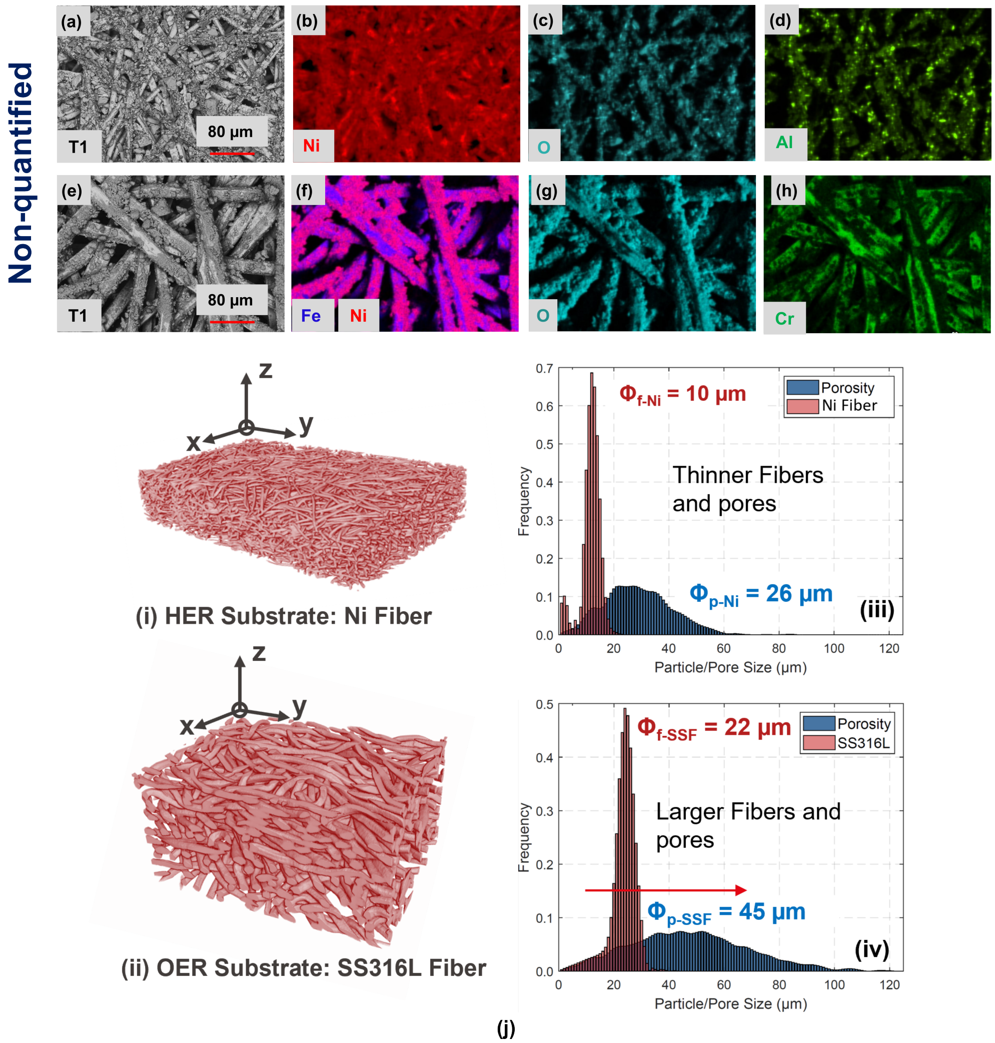

Figure S4: **(a)** SEM images of commercial catalyst supported substrate electrodes with Raney® nickel on nickel fibre paper as a HER-PTL layer, and **(b)** the EDX intensity map showing nickel rich substrate and along with the presence of **(c)** oxygen and **(d)** aluminium. **(e)** $NiFe_2O_4$ on SS316L fibre paper as OER-PTL layer, **(f)** the EDX intensity map showing nickel-iron rich surface along with **(h)** oxygen and **(h)** substrate chromium. **(j)** The 3D-reconstruction of the (i) HER-PTL and (ii) OER-PTL and the corresponding phase distributions (iii) and (iv) respectively.

lattice parameter of 3.525 Å. XRD analysis of the HER electrode in Fig. S5a did not detect the expectant planes associated with the material NiFeCo, only those associated with nickel.

Conversely, the anode XRD spectra of $NiFe_2O_4$ on SS316L substrate showed the expectant spinel peaks at 18.54° 30.48°, 35.80°, 54.29°, 57.56° corresponding to 111, 220, 311, 422 and 511 reflection planes respectively [65, 66], as shown in Fig. S5b. Additionally, traces of the stable α-Fe_2O_3 (hematite) where found at 33.32° and 63.14°, affiliated to 104 and 300 planes respectively [67, 68]. The strong peaks at 43.69°, 50.87°, and 74.74° represents the planes 111, 200, and 220 from austenitic stainless steel [28, 69]. The corresponding Rietveld refinement confirmed the spinel Fd-3m Z (227) with 8.339(2) Å [70].

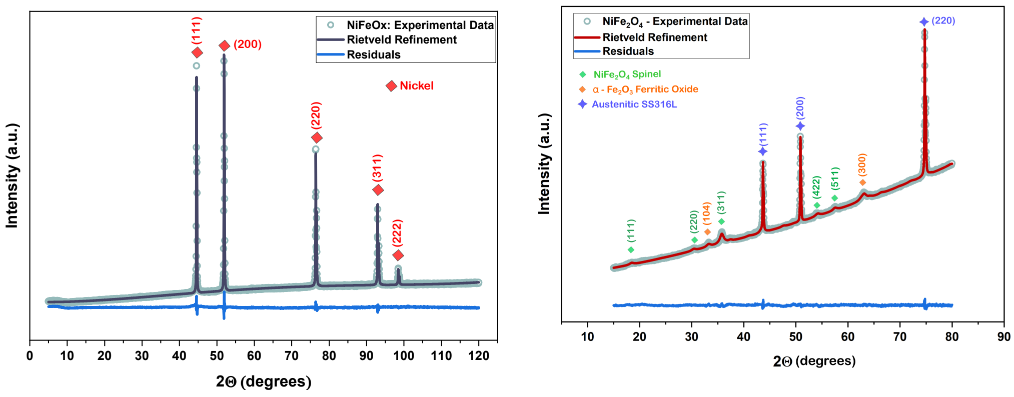

(a)

(b)

Figure S5: The Rietveld refined XRD spectra of **(a)** Raney® nickel on nickel fibre HER electrode and **(b)** the $NiFe_2O_4$ on SS316L fibre OER electrode.

XPS was conducted to quantify the pristine oxidation state of the electrodes. Given the unexpected cathode XRD spectra, XPS survey were collected for the $NiFe_2O_4$ anode and NiFeCo cathode and compared to comparable catalyst powders purchased from US Research Nanomaterials as shown in Fig. S6. The anodic catalyst materials in Fig. S6a display similar peaks, though with the obvious difference originating in the Nafion binder used for the anode electrode. Conversely, the cathode electrode supplied by Dioxide Materials did not appear to contain cobalt, as its associated 2p and 3p peaks were not found, which is easily seen in comparison with the powder reference in Fig. S6b. Additionally, it was difficult to determine whether iron was present,

as the iron 2p peaks coincide with the fluorine 1s relaxation peak. As such, a detailed investigation of the core-level spectra was necessary. However, it is clear from the XRD- and XPS survey-spectra that the Dioxide Materials cathode is not NiFeCo, but likely a Raney-nickel type catalyst material.

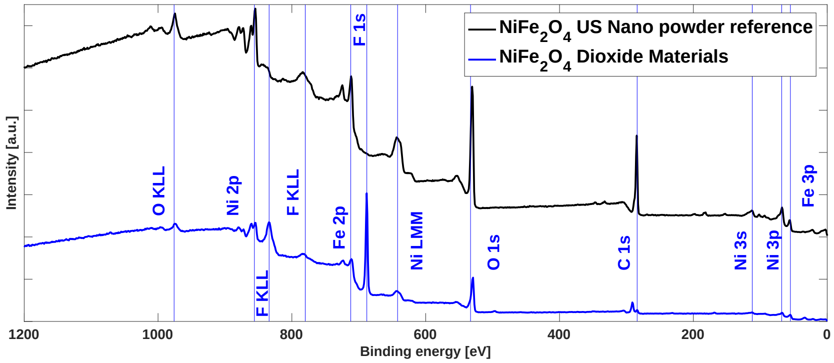

(a)

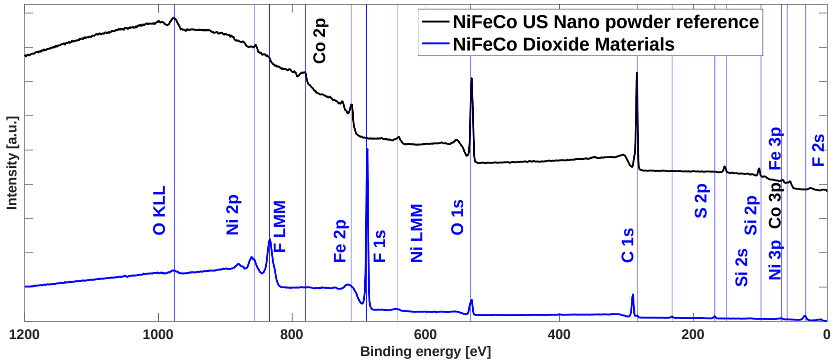

(b)

Figure S6: XPS survey spectra comparing **(a)** the commercial anode material from Dioxide Materials against a comparable powder reference bought from US Research Nanomaterials and likewise so for the **(b)** cathode material.

Considering Nafion perfluorinated resin solution was employed as a binder for both electrodes, its influence in complicating the analysis was considerable. As previously specified, the binding energy affiliated with the fluorine 1s relaxation peak and the fluorine KLL Auger peak coincides with the 2p spectra of iron and nickel respectively. As such, we gathered additional core-level spectra from the fluorine 1s relaxation peak and the fluorine KLL

Auger peak for pure Nafion perfluorinated resin solution (Fig. S7), which were compared against the anode and cathode. Here, both the fluorine 1s relaxation peak and iron 2p affect one region, and both the fluorine KLL Auger peak and the nickel 2p affect another. Thus, an accurate estimate of the influence of fluorine was gained through comparative analysis where a good fit was achieved when accounting for both fluorine and 2p metal influence. Presence of fluorine in the iron and nickel 2p spectra is clearly shown in Fig. S8a - S8b and Fig. S8d - S8e.

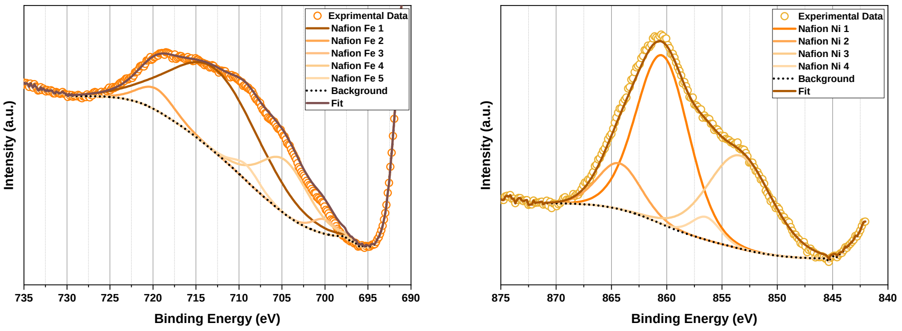

(a) (b)

Figure S7: The XPS features of Nafion 117 in the **(a)** Fe $2p_{3/2}$ region and the **(b)** Ni - $2p_{3/2}$ region.

The survey spectrum shows several distinct peaks, all related to the components of the catalyst layer, namely Raney nickel and Nafion resin. After accounting for the influence of Nafion, it was clear that iron was not present. Despite the superimposing fluorine KLL Auger peak, the nickel $2p_{3/2}$ spectrum around 860 eV could be analysed and decomposed into contributions from NiO, $Ni(OH)_2$ and NiOOH as shown in Fig. S8a, with peak binding energies at 855.26, 856.16 and 858.11 eV respectively. An average oxidation state slightly greater than +2 due to the presence of NiOOH, corresponding to slightly oxidised Raney nickel [64].

Appraising the affiliated oxygen 1s spectrum at 531 eV reveals contributions at 529.85 eV, 531.83 eV, 533.8 eV as illustrated in Fig. S8c. The initial peak originates from the M-O_x bond of the various nickel oxides [64]. The small metal oxide contribution matches the slightly increased oxidation state indicated by the nickel 2p spectrum. Given the great amount of Nafion resin in the catalyst layer, the dominant contribution at 531.83 eV likely originates

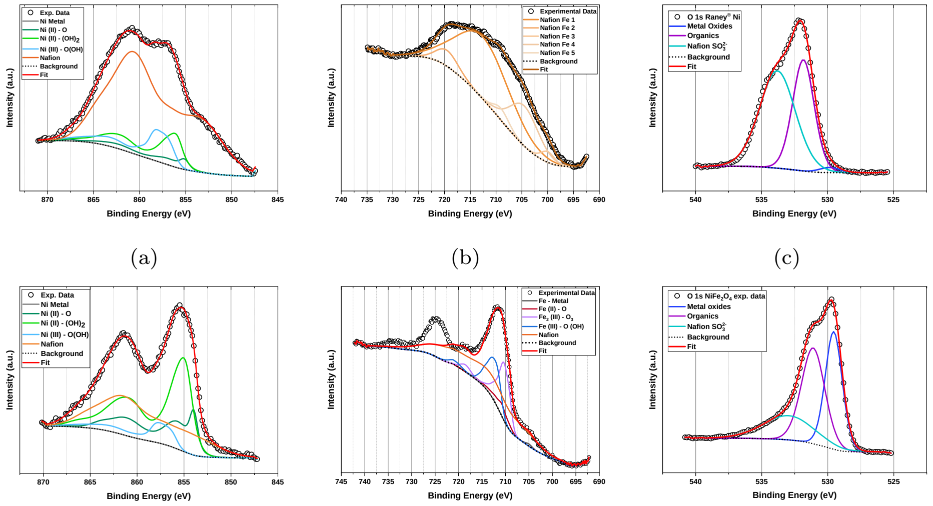

(a) (b) (c)

(d) (e) (f)

Figure S8: The XPS spectra for HER Raney® nickel **(a)** nickel $2p_{3/2}$ and **(b)** iron $2p_{3/2}$ spectra with **(c)** the oxygen 1s. The XPS spectra for the OER $NiFe_2O_4$ shown for **(d)** nickel $2p_{3/2}$ and **(e)** iron $2p_{3/2}$ spectra with the **(f)** oxygen 1s spectrum.

from various organic bonds (O-CF, O-CF_2), and the final peak at 533.8 eV from the -SO_3, the charge carrying group of Nafion resin [71]. Additionally, an oxygen 1s spectrum was measured for pure Nafion resin, which indicated three contributions, around 537.15, 534.58 and 532.62 eV. These are affiliated with adsorbed water, the –C_2–O-CF_2– bond and the -SO_3 bond [71]. Compared against the cathode, the Nafion content was significantly lower by ≈ 40 wt.% ($\frac{(F\ \ 1s)_{anode}}{(F\ \ 1s)_{cathode}} = 0.6036$), where this was also reflected in the carbon 1s spectrum due to the PTFE originating C-F_2 bond ($\frac{(C{-}F_2)_{anode}}{(C{-}F_2)_{cathode}} = 0.5739$).

The nickel $2p_{3/2}$ spectrum of the $NiFe_2O_4$ anode was deconvoluted to show contributions from NiO, $Ni(OH)_2$ and NiOOH as shown in Fig. S8d, with peak binding energies at 853.95, 855.0 and 857.55 eV respectively. The average oxidation state was marginally above +2, agreeing with the theoretical oxidation numbers in $NiFe_2O_4$ [72, 73]. The iron $2p_{3/2}$ spectrum was deconvoluted to reveal components from metallic Fe, FeO, Fe_2O_3 and FeOOH, with peak binding energies at 704.95, 710.80, 710.35 and 712.15 eV respectively (Fig. S8e). The average oxidation state is slightly below +3, which fits the expected oxidation state from the originating spinel structure [72, 73]. Fig. S8f exhibits the anodic oxygen 1s spectrum at 529 eV, showing supporting

evidence for the Ni- and Fe-spectra, with contributions at 529.55 eV, 531.10 eV and 532.94 eV. The dominant peak at 529.55 eV comes from the lattice oxygen in the anode catalyst. The peaks at 531.10 eV and 532.94 eV are likely from organic bonds (e.g. $-SO_3$ charge carrier) in the Nafion ionomer and the O-CF, $O-CF_2$ bonds, respectively [71]. Nafion-related O 1s peaks are both shifted down by $\sim$0.8 eV relative to the cathode, despite both anode and cathode showing the same binding energies for their $C-F_2$ peaks remaining constant in both C 1s and F 1s spectra. Their $-SO_3$ peaks also remain at the same positions as seen through the S 2p spectra, and these trends are also seen in the survey spectra. However, the cathode spectra are align qualitative with that of the pure Nafion resin, implying the that the $-SO_3$ lies buried in the peak for organic bonds without being able to conclusively separate a single contribution. This would also indicate a slight reduction of the Nafion on the cathode, which is likely considering it comprises Raney nickel which is easily oxidised.

Table S3: The nickel oxidation state comparison between the HER and OER electrocatalysts obtained through XPS.

**Catalyst**	**Ni - Metal (at.%)**	**Ni (II) - O (at.%)**	**Ni (II) - $(OH)_2$ (at.%)**	**Ni (III) - O (OH) (at.%)**
**HER: Raney® nickel**	0.00	15.01	45.29	39.70
**OER: $NiFe_2O_4$**	0.03	27.92	60.22	11.83

Electrochemical

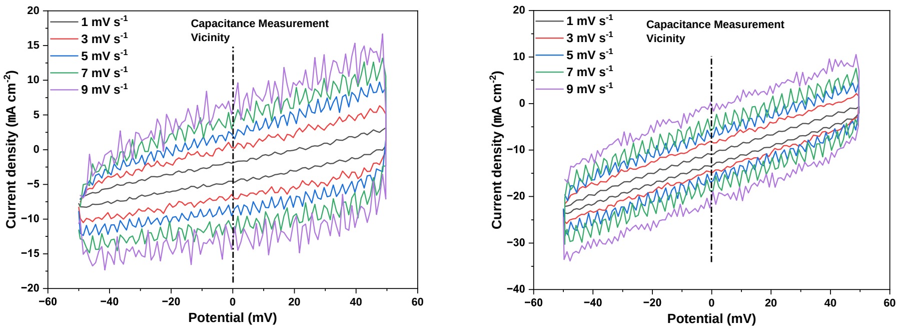

(a) (b)

Figure S9: The double layer capacitance of **(a)** the Raney® nickel HER electrocatalyst and **(b)** $NiFe_2O_4$ OER electrocatalyst

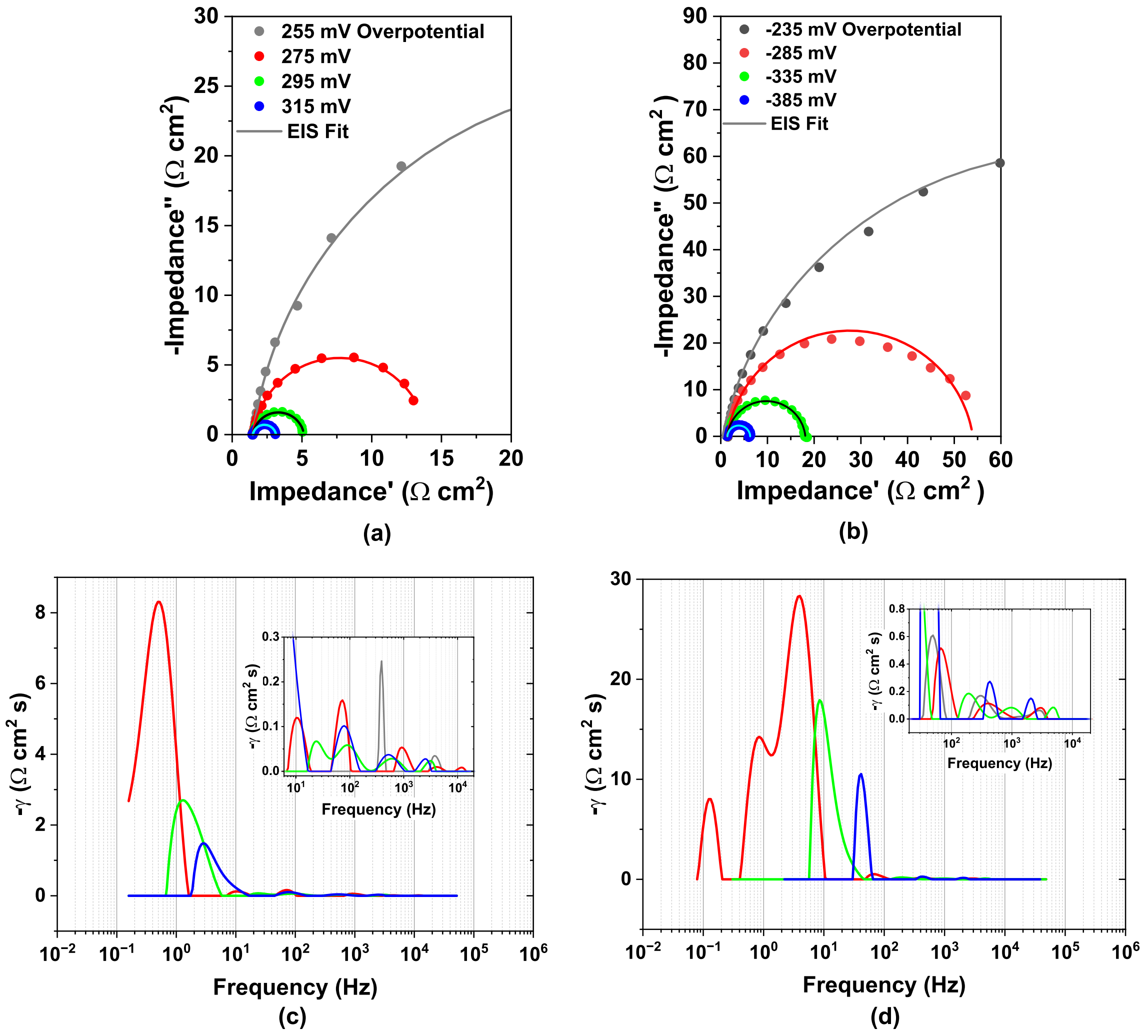

Figure S10: Nyquist fit results with 3 electrode setup at 20 °C. **(a)** OER and **(b)** HER, respectively. The corresponding DRT data, with zoomed insets for **(c)** OER and **(d)** HER.

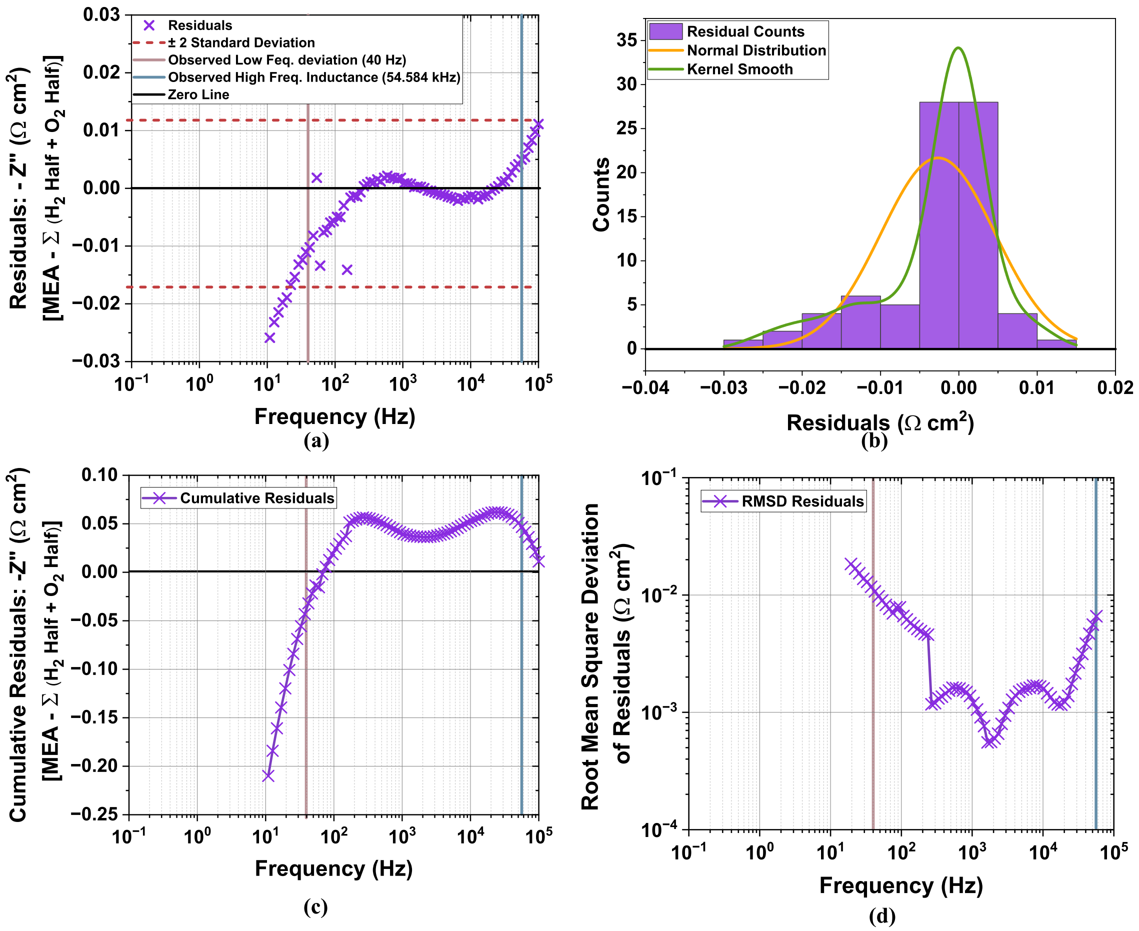

Figure S11: **(a)** The residual plot of the sum of half-cell EIS measurement with respect to MEA EIS data at 0.5 A cm^{-2}, 20 °C. ±2 SD indicates a statistically acceptable window of data points for analysis, while the frequency windows represent an electrochemically acceptable window. **(b)** The corresponding histogram shows the distribution of residuals. **(c)** The cumulative residuals showing deviations at low and high frequencies. **(d)** The root mean square deviation of residuals value plotted in a log-log graph showing low deviation.

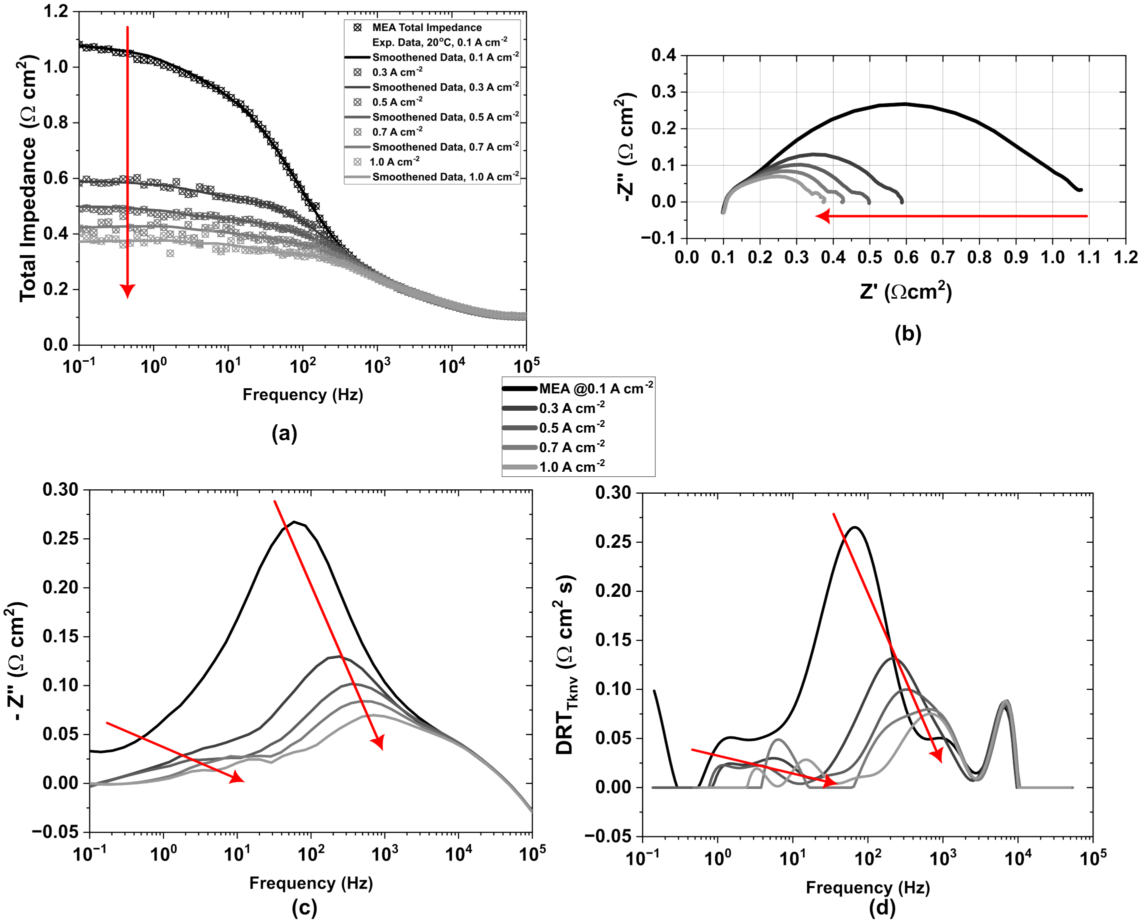

Figure S12: **(a)** The raw and the smoothened MEA EIS data as a function of current density, at 20°C, 1 M KOH. **(b)** The corresponding Nyquist and **(c)** Imaginary impedance plots of the smoothened data. **(d)** The MEA distribution of relaxation times plot, highlighting electrochemical and physical process change trends.

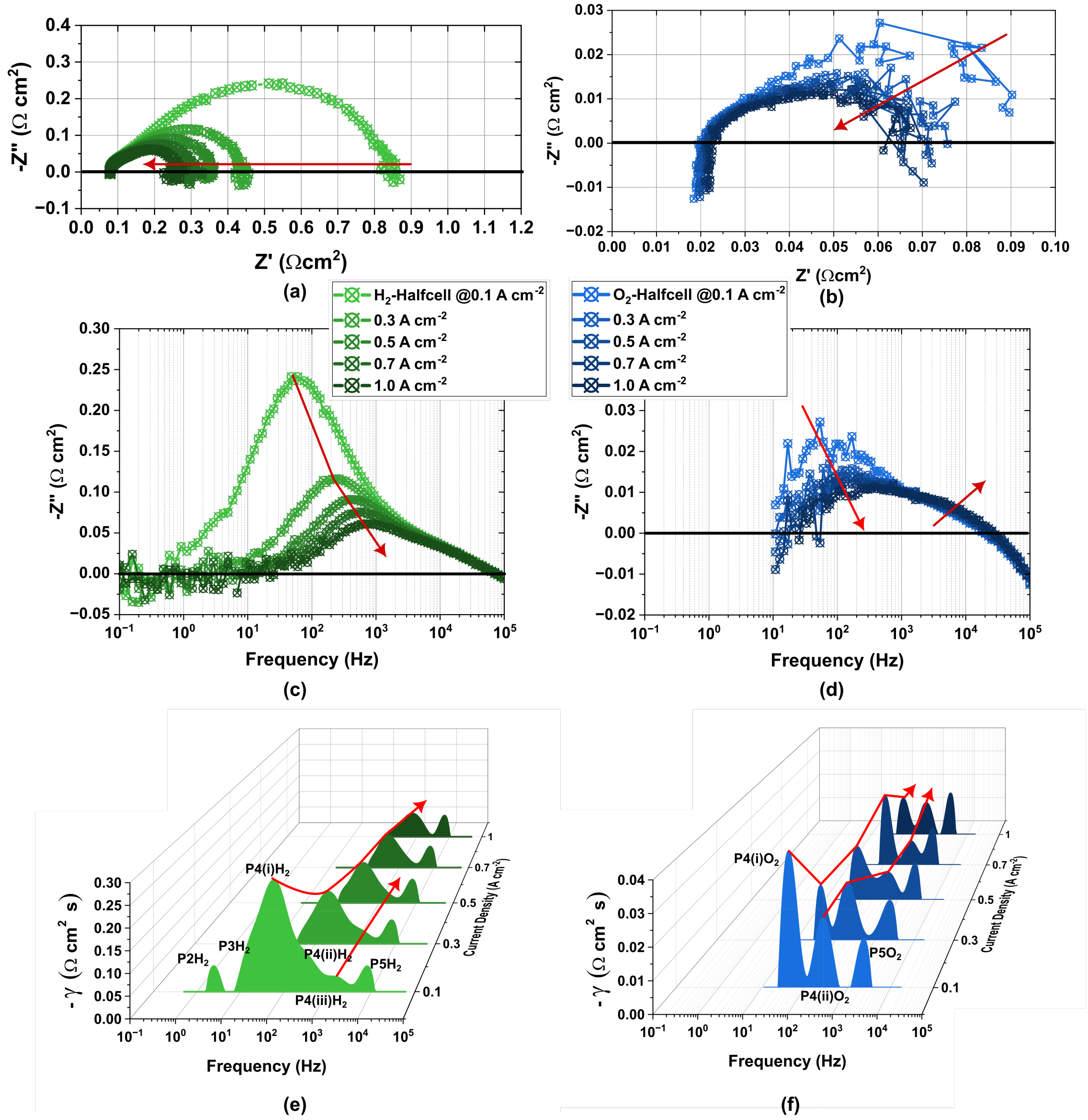

Figure S13: The EIS measurements as a function of increasing current density for H_2 and O_2 electrode-reference electrode system.

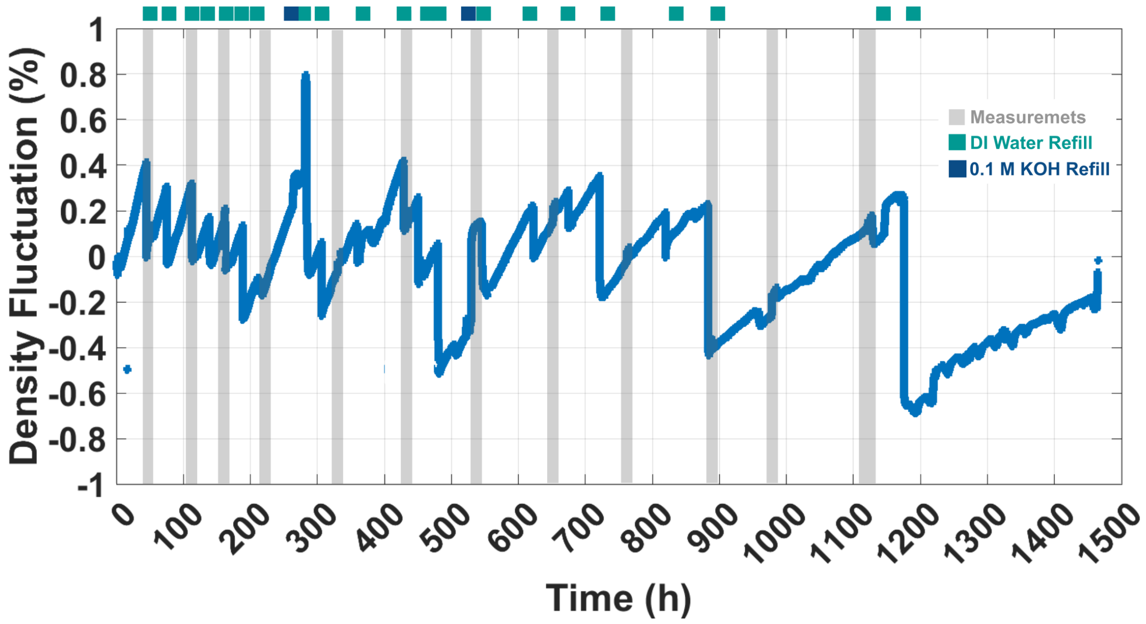

Figure S14: The fluctuation in the density due to MEA operation and refill.
